# The efficacy and safety assessment of oncolytic virotherapies in the treatment of advanced melanoma: a systematic review and meta-analysis

**DOI:** 10.1186/s12985-023-02220-x

**Published:** 2023-11-02

**Authors:** Changyuan Wang, Nanxiao Lu, Lin Yan, Yang Li

**Affiliations:** Department of Dermatology, University of Health and Rehabilitation Sciences (Qingdao Municipal Hospital), NO.1 Jiaozhou Road, Qingdao, 266000 Shandong Province China

**Keywords:** Virotherapy, Oncolytic virus, Advanced melanoma, Adverse events, Treatment response, Overall survival, Progression-free survival

## Abstract

**Background:**

The efficacy and safety of oncolytic virotherapies in the treatment of advanced melanoma still remains controversal. It is necessary to conduct quantitative evaluation on the basis of preclinical trial reports.

**Methods:**

Publicly available databases (PubMed, Embase, Medline, Web of Science and Cochrane Library.) and register (Clinicaltrials.gov) were searched to collect treatment outcomes of oncolytic virotherapies (including herpes simplex virus type 1 (HSV), coxsackievirus A21 (CVA21), adenovirus, poxvirus and reovirus) for advanced/unresectable melanoma. Comparisons of treatment response, adverse events (AEs) and survival analyses for different virotherapies were performed by R software based on the extracted data from eligible studies.

**Results:**

Finally, thirty-four eligible studies were analysed and HSV virotherapy had the highest average complete response (CR, 24.8%) and HSV had a slightly higher average overall response rate (ORR) than CVA21 (43.8% vs 42.6%). In the pooled results of comparing talimogene laherparepve (T-VEC) with or without GM-CSF/ICIs (immune checkpoint inhibitors) to GM-CSF/ICIs monotherapy suggested virotherapy was more efficient in subgroups CR (RR = 1.80, 95% CI [1.30; 2.51], P < 0.01), ORR (RR = 1.17, 95% CI [1.02; 1.34], P < 0.05), and DCR (RR = 1.27, 95% CI [1.15; 1.40], P < 0.01). In patients treated with T-VEC+ICIs, 2-year overall survival (12.1 ± 6.9 months) and progression-free survival (9.9 ± 6.9) were significantly longer than those treated with T-VEC alone. Furthermore, we found that AEs occurred frequently in virotherapy but decreased in a large cohort of enrolled patients, some of which, such as abdominal distension/pain, injection site pain and pruritus, were found to be positively associated with disease progression in patients treated with T-VEC monotherapy.

**Conclusion:**

Given the relative safety and tolerability of oncolytic viruses, and the lack of reports of dose-limiting-dependent toxicities, more patients treated with T-VEC with or without ICIs should be added to future assessment analyses. There is still a long way to go before it can be used as a first-line therapy for patients with advanced or unresectable melanoma.

**Supplementary Information:**

The online version contains supplementary material available at 10.1186/s12985-023-02220-x.

## Introduction

Patients with advanced melanoma had a poor prognosis with surgery and chemotherapy, until immune checkpoint inhibitors (ICIs) made breakthroughs in tumor regression and long-term durable cancer control [1–3]. However, occurrence of immune-related adverse events and a cold tumor microenvironment result in poor response and drug resistance over time [4]. Therefore, the induction of “cold tumors” into “hot tumors” has become the target of the next generation of antitumour therapy [5, 6]. Oncolytic viruses (OVs), which stimulate host antitumour immunity by preferentially replicating in tumor cells and forming a hot tumor environment, are a highly favorable tumor therapy strategy for patients with poor response to ICIs [7, 8]. Since virotherapy with talimogene laherparepvec (T-VEC) was approved for use in the United States and Europe, it has been explored in preclinical treatment studies for advanced/unresectable melanoma for nearly a decade [9–11]. Numerous clinical trials in advanced melanoma have evaluated the efficacy of T-VEC and other novel OVs (e.g. reovirus, poxvirus, etc.) in combination with immunosuppressants, including ipilimumab, nivolumab, and pembrolizumab [12–14]. There have been several reviews on the efficacy evaluation of virotherapy for advanced or metastatic melanoma [15–17], however, the quantitative evaluation of different oncolytic virotherapies has not been reported. Therefore, on the basis of data on treatment response, adverse events and survival, this study conducted a systematic meta-analysis to evaluate the efficacy and safety of different OVs and to promote our understanding of virotherapies in advanced melanoma.

## Materials and methods

### Searching strategies

We searched the following databases from January 1, 2000 to December 31, 2022: PubMed, Embase, Medline, Web of Science, Cochrane Library and online Clinicaltrials.gov. The keywords used in the search included “oncolytic virus”, “advanced/metastatic/unresectable melanoma”, “adverse event”, “overall survival”, “talimogene laherparepvec”, “herpesvirus”, “reovirus”, “coxsackievirus”, “poxvirus” and “adenovirus”. For example, the search formulas were ((oncolytic virus [Title/Abstract]) OR (talimogene laherparepvec) OR (poxvirus) OR (herpesvirus) OR (reovirus) OR (coxsackievirus)) AND ((advance melanoma [Title/Abstract]) OR (metastatic melanoma)), ((virotherapy [Title/Abstract]) AND (melanoma [Title/Abstract])) AND ((adverse event [Title/Abstract]) OR (overall survival [Title/Abstract])).

### Inclusion and exclusion criteria

Inclusion criteria: (a) The recruited patients have unresectable, advanced or metastatic melanoma; (b) The virotherapies are limited to human herpes simplex virus type 1 (HSV), coxsackievirus A21 (CVA21), reovirus, adenovirus and poxvirus; (c) The treatment responses were assessed by the response evaluation criteria for solid tumors (RECIST), including complete response (CR), partial response (PR), stable disease (SD), progressive disease (PD) and adverse events (AEs) (optional); (d) The study types are limited to clinical trials, retrospective or randomized control trials (RCTs); (e) The study design described detailed treatment procedures.

Exclusion criteria: (a) Patients suffer from active cerebral, bone, or any more than 3 visceral metastases; (b) The recruited patients had an early stage (I-II) of the disease; (c) The recruited patients were younger than 18 years of age; (d) Data and results have been reported in previous studies; (e) Clinical trials are ongoing, and the results there are not yet available; (f) Animal studies and in vitro studies.

### Data collection

Three staff members (CY.W, NX.L and L.Y) were independently involved in data extraction and rectification, including database retrieval, duplicate examination and treatment endpoint recording. Subsequently, full-text publications and registers were reviewed against eligibility criteria and differences were resolved by discussion and mutual consent with the two investigators (CY.W and NX.L). For eligible studies, the following were extracted independently by two researchers (NX.L and L.Y) using the same format table: name of first author, year of publication, age (mean/range) of enrolled patients, phase of clinical trial, treatment arms of study, viral family of OV, and administration of study drug. Survival data of enrolled patients could not be obtained directly from the original studies. Therefore, we used the Engauge Digitizer tool (version 12.1, https://github.com/markummitchell/engauge-digitizer) to digitize the overall survival (OS) and progression-free survival (PFS) curves provided in the publications. All curves were then redrawn and compared in the OS or PFS composite graphs.

### Quality assessment

The quality assessment of RCTs was evaluated by two researchers (NX.L and Y.L) using the Cochrane Collaboration's tool [18], including seven items. Each item consists of three risk levels: low, high and unclear. Other studies were evaluated with 8 items of the scale of methodological index for non-randomized studies [19]. Details of the quality assessment results were attached to the Additional file 3.

### Statistical analysis

All statistical analyses were conducted by R software (version 4.1.3, Copyright (C) 2022 The R Foundation for Statistical Computing). The combination method for single-sample proportions follows the guidance in the R package meta (version 5.2.0) described by Balduzzi [20]. The overall response rate (ORR) and disease control rate (DCR) were calculated based on the events of CR, PR and SD: ORR = CR+PR and DCR = ORR+SD. The relationship between treatment response and AEs was calculated by the Pearson correlation test. In single-arm studies, Freeman-Tukey Double arcsine transformation was used to polled an overall inverse-variance (IV). In binary data of some multi-arm studies, the IV method was used to combine and compare the estimated effect sizes. The risk ratio (RR) and P value less than 0.05 were used as the criteria of significance.

## Results

### Basic information of included studies

According to the flow diagram of databases and registers retrieval and screening described in PRISMA(2020) [21] (Additional file 1: Fig. S1). Finally, 21 clinical trials (Table 1) (4 of which did not provide publications [22–25]) and 10 other studies [13, 14, 26–33] were included in this analysis. Clinical trials of NCT02263508 [34, 35], NCT00769704 [36, 37] and NCT01740297 [38, 39] reported conclusions twice with different sample sizes until completion. Six of these clinical trials [40–45] did not post results on ClinicalTrials.org at the time of completion of this study (Table 1). A total of 2,710 patient records were used to assess the efficacy and safety of virotherapies.

**Table 1:** Characteristics of included studies

Author (Year) [Ref.]	Phase	Median/Mean age (range)	Viral family	Treatment drugs (N)	End Points	Registered ID	Study drug administration	Substages
Shoushtari AN (2022) [12]	I	73(40–87)	Adenovirus	ONCOS-102+Pembrolizumab (20)	TR (DCR: 55%; ORR: 35%), AE	NCT03003676	Part 1: 3 doses of IT injection of ONCOS-102 (days 1, 4, and 8) at 3 × 10^11^ viral particles (VP), preceded by IV cyclophosphamide priming 1–3 days prior to day 1. Then receive pembrolizumab IV, 2 mg/kg or 200 mg flat dose on day 22 (Week 3) and every three weeks thereafter until Day 169/Week 24 Part 2: 4 doses of NCOS-102 (Days 1, 4, 8, and 15) followed by 8 doses of ONCOS-102 plus pembrolizumab every 3 weeks	III: 11(52%) IVM1a: 4(19%) IVM1b: 2(10%) IVM1c: 4(19%)
NCT03259425 (2022) [22]	II	71(18–83)	HSV	HF10 + Nivolumab (7)	TR (DCR: 57.1%; ORR: 42.9%), AE	NCT03259425	HF10: 1 × 10^7^th TCID50/mL, IT injection to a single or multiple eligible tumors for a total of 5 mL; on days 0, 7, 14, 21, 28, 42, 56, 70, 84 for a total of 9 injections Nivolumab: a dose of 240 mg given as an IV infusion on day 0. Every 14 days for a total of 7 infusions. Then at a flat dose of 480 mg IV every 28 days for up to one year	IIIB, IIIC, or IVM1a
Cui C (2022) [46]	Ib	59(26–83)	HSV	OrienX010 (26)	TR (DCR: 53.8%; ORR: 19.2%), OS, PFS, AE	NCT01935453^¶^	Cohort 08 received IT injections of OrienX010 up to 5 mL of 8 × 10^7^ PFU/mL every 2 weeks. Patients received IT injections of OrienX010 up to 10 mL of 8 × 10^7^ PFU/mL every 2 weeks. Cohort 09 received IT injections of OrienX010 up to 10 mL of 8 × 10^7^ PFU/mL every 2 weeks	IIIC: 9(34.6%) IVM1a: 11(42.3%) IVM1b: 5(19.2%) IVM1c: 1(3.8%)
Chesney J (2022) [35]	III	Arm1: 63.1 ± 13.7 Arm2: 62.3 ± 14.5	HSV	Arm1: T-VEC + Pembrolizumab (346) Arm2: Placebo+Pembrolizumab (346)	TR (DCR: 56.6% v 50%; ORR: 48.6% v 41.3%), OS, PFS, AE	NCT02263508^†^ (MASTERKEY-265)	Participants received T-VEC/placebo at an initial dose of up to 4 mL 10^6^ PFU/mL by intralesional injection on day 1. Subsequent doses of T-VEC/placebo at 10^8^ PFU/mL (up to 4 mL) began 3 weeks after the first dose and were administered every 2 weeks until the fifth injection of T-VEC (week 9), and then synchronously with pembrolizumab thereafter every 3 weeks until disappearance of injectable lesions, CR, etc	IIIB: 18(5.2%) v 20(5.8%) IIIC: 66(19.1%) v 53(15.3%) IVM1a: 69(19.9%) v 81(23.4%) IVM1b: 48(13.9%) v 49(14.2%) IVM1c: 145(41.9%) v 143(41.3%)
Schwarze JK (2022) [40]	I	64(31–81)	HSV	T-VEC (12)	TR (DCR: 41.7%; ORR: 25%), AE	NCT03747744^¶^	On day 1, IT injections of selected lesions with T-VEC 10^6^ PFU/mL (up to 4 mL). Then treated with T-VEC (10^8^ PFU/mL, up to 4 mL) on day 21 and every 14 days thereafter until CR, PD, unacceptable toxicity, etc	IIIC: 1(8%) IVM1a: 3(23%) IVM1b: 1(8%) IVM1c: 6(46%) IVM1d: 2(15%)
Andtbacka RHI (2021) [47]	II	64(28–94)	CVA21	V937 (57)	TR (DCR: 38.6; ORR: 28.1%), OS, PFS, AE	NCT01227551; NCT01636882	IT injection at a dose of 3.3 × 10^8^ TCID_50_ in a volume of 4 mL Once a day on days 1, 3, 5, 8, 22, 43, 64, 85, 106, and 127	IIIC: 22(38.6%) IVM1a: 14(24.6%) IVM1b: 9(15.8%) IVM1c: 12(21.1%)
Dummer R (2021) [10]	II	62.6(12.6)	HSV	T-VEC (76)	TR (DCR: 40.8%; ORR: 13.2%), OS, PFS, AE	NCT02211131	IT injection at a dose of 10^6^ PFU/mL (up to 4 mL) followed by 10^8^ PFU/mL 3 weeks later and every 2 weeks thereafter until week 12	IIIB/C: 58(76.3%) IV: 17(22.4%)
Malvehy J (2021) [9]	II	68(26–90)	HSV	T-VEC (111)	TR (DCR: 39.6%; ORR: 20.7%), OS, AE	NCT02366195	IT injection at an initial dose of 10^6^ PFU/mL on day 1 followed by a dose of 10^8^ PFU/mL 21 days after the initial dose and every 14 days thereafter	IIIB: 14(12.6%) IIIC: 32(28.8%) IVM1a: 38(34.2%) IVM1b: 15(13.5%) IVM1c: 12(10.8%)
Ressler JM (2021) [26]	NA	72(36–95)	HSV	T-VEC (88)	TR (DCR: 72.7%; ORR: 63.6%), OS, PFS, AE	NA	The initial dose on day 1 was 10^6^ PFU/mL (up to 4 mL). The second dose on day 21 was 10^8^ PFU/mL (up to 4 mL), the following cycles were applied every 14 days thereafter with 10^8^ PFU/mL	IIIB: 9(10.2%) IIIC: 47(53.4%) IIID: 1(1.1%) IVM1a: 18(20.5%) IVM1b: 5(5.7%) IVM1c: 4(4.5%) IVM1d: 4(4.5%)
Stahlie EHA (2021) [41]	NA	69(30–97)	HSV	T-VEC (93)	TR (ORR: 79.6%), PFS, AE	NCT04330430^¶^	At an initial dose of 10^6^ PFU/mL and all doses thereafter at 10^8^ PFU/mL. Repeats every 2 weeks, the maximum injection volume per treatment session is 4.0 ml and the volume that is injected depends on the size of the lesion(s)	IIIB: 30(32.3%) IIIC: 56(60.2%) IIID+IVM1a: 7(7.5%)
Andtbacka RHI (2019) [11]	II	65(19–93)	HSV	T-VEC (60)	TR (DCR: 76.3%; 31.5%), OS, AE	NCT02014441	IT injection at an initial dose of 10^6^ PFU/mL, 10^8^ PFU/mL 21 days later, and 10^8^ PFU/mL every 14 (± 3) days thereafter	IIIB: 10(17%) IIIC: 32(53%) IVM1a: 11(18%) IVM1b: 3(5%) IVM1c: 4(7%)
García M (2019) [42]	I	51(40–80)	Adenovirus	ICOVIR-5 (12)	TR (DCR: 58.3%), OS, AE	NCT01864759^¶^	Administered as a single infusion, IV dose levels: 1a, 1 × 10^11^ viral particles (vp); 2a, 3.3 × 10^11^ vp; 3a, 1 × 10^12^ vp; 4a, 3.3 × 10^12^ vp; and 5a, 1 × 10^13^ vp. A dose level of 1 × 10^10^ (-1a) was planned if dose 1a became limiting	IVM1a: 5(41.7%) IVM1c: 6(50%) IVM1d: 1(8.3%)
Louie RJ (2019) [28]	NA	68.8(33–94)	HSV	T-VEC (80)	TR (DCR: 81.3%; ORR: 56.3%),OS, AE	NA	At an initial dose of 10^6^ PFU/mL (up to 4 mL). Three weeks after, at dose of 10^8^ PFU/mL, repeated q2w until CR, etc	IIIB: 37(46.3%) IIIC: 25(31.3%) IIID: 1(1.3%) IV: 16(20%)
Franke V (2019) [27]	NA	73.9(37.9–87.9)	HSV	T-VEC (26)	TR (DCR: 92.3%; ORR: 82.5%), AE	NA	At an initial dose of 10^6^ PFU/mL. Three weeks after, at dose of 10^8^ PFU/mL, and thereafter once every 2 weeks. Total volume per treatment session is ≤ 4.0 mL	IIIB: 14(53.8%) IIIC: 12(46.2%)
Andtbacka RHI (2019) [36, 48]	III	Arm1: 63.07 ± 13.68 Arm2: 62.92 ± 14.13	HSV	Arm1: T-VEC (295) Arm2: GM-CSF (141)	TR (DCR: 76.3% v 56.7%; ORR: 31.5% v 6.4%), OS, AE	NCT00769704^†^ (OPTiM)	Arm1: IT injection at an initial dose was at 10^6^ PFU/mL. At least 3 weeks after the first dose and consisted of T-VEC at a concentration of 10^8^ PFU/mL. On days 1 and 15 of each 28-day cycle for 24 weeks Arm2: GM-CSF was administered at a dose of 125 μg/m^2^/day subcutaneously for 14 days in 28-day cycles for 24 weeks	IIIB: 22(7.5%) v 12(8.5%) IIIC: 66(22.3%) v 31(22.0%) IVM1a: 76(25.8%) v 43(30.5%) IVM1b: 64(21.7%) v 26(18.4%) IVM1c: 67(22.7%) v 29(20.6%)
Sun L (2018) [29]	NA	70(52–82)	HSV	T-VEC+anti-PD-1 (10)	TR (ORR: 90%), OS, PFS, AE	NA	T-VEC was administered on day 1 of weeks 4 and 6 and every 2 weeks thereafter. Refer to protocol in MASTERKEY-265	III: 8(80%) IV: 2(20%)
Chesney J (2018) [38]	II	Arm1: 65(23–93) Arm2: 64(23–90)	HSV	Arm1: T-VEC + Ipilimumab (98) Arm2: Ipilimumab (100)	TR (DCR: 58.2% v 32%; ORR: 38.8% v 18%), OS, PFS, AE	NCT01740297^†^	Arm1: IT injection at an initial dose of 10^6^ PFU/mL (up to 4 mL). Three weeks later, a doses at 10^8^ PFU/mL q2w until CR, etc. Then received 3 mg/kg ipilimumab IV q3w for a total of 4 infusions starting at the time of the third dose of T-VEC (week 6) Arm2: Ipilimumab: 3 mg/kg IV q3w for a total of 4 infusions starting at week 1	IIIB: 5(5%) v 9(9%) IIIC: 29(30%) v 31(31%) IVM1a: 16(16%) v 17(17%) IVM1b: 20(20%) v 10(10%) IVM1c: 28(29%) v 33(33%)
Perez MC (2018) [30]	NA	75(51–94)	HSV	T-VEC (23)	TR (DCR: 78.3%; ORR: 56.5%), OS	NA	IT injection at an initial dose of 10^6^ PFU/mL (up to 4 mL). Three weeks later, a second injection cycle at a dose of 10^8^ PFU/mL, q2w until either PD, CR, etc	IIIB: 9(33%) IIIC: 13(48%) IVM1a: 4(15%) IVM1c: 1(4%)
Mahalingam D (2017) [13]	II	56(23–78)	Reovirus	REOLYSIN® +paclitaxel/carboplatin (14)	TR (DCR: 85.7%; ORR: 21.4%), OS, PFS, AE	NA	IV injection paclitaxel at a dose of 200 mg/m^2^ on day 1 of each cycle, followed by REOLYSIN® IV at a dose of 3 × 10^10^ TCID_50_. On days 2–5, REOLYSIN® was administered alone using the same dose on day 1. The treatment cycles were repeated every 21 days for up to 8 cycles	NA
Ribas A (2017) [34]	Ib	58(37–89)	HSV	T-VEC+Pembrolizumab (21)	TR (DCR: 76.2%; ORR: 71.4%), OS, PFS, AE	NCT02263508^†^	IT at an initial dose of up to 4 mL 10^6^ PFU/mL on day 1 of week 1. 3 weeks after, at up to 4 mL of 10^8^ PFU/mL q2w until disappearance of injectable lesions,CR, etc	IIIB: 1(4.8%) IIIC: 6(28.6%) IVM1a: 2(9.5%) IVM1b: 4(19.0%) IVM1c: 8(38.1%)
Puzanov I (2016) [39]	Ib	61(29–84)	HSV	T-VEC+Ipilimumab (19)	TR (DCR: 68.4%; ORR: 47.4%), OS, PFS, AE	NCT01740297^†^	IT injection at an initial dose of 10^6^ PFU/mL (up to 4 mL total). 3 weeks after, at 10^8^ PFU/mL (up to 4 mL total), q2w. IV ipilimumab 3 mg/kg q3w for a total of 4 infusions starting at the time of the third dose of T-VEC (week 6)	IIIB: 1(5%) IIIC: 3(16%) IVM1a: 4(21%) IVM1b: 5(26%) IVM1c: 6(32%)
Curti B (2016) [43]	Ib	18 Years and older	CVA21	CAVATAK™(CVA21)+Ipilimumab (7)	TR (DCR: 85.7%; ORR: 57.1%)	NCT02307149^¶^	IT injection up to 3 × 10^8^ TCID_50_ on study days 1, 3, 5, 8 and 22, and then q3w for a further 6 series of injections. Ipilimumab (3 mg/kg) q3w was given as 4 i.v. infusions starting at Day 22	IIIC-IVM1c
Kaufman HL (2016) [31]	II	Ref. Senzer NN	HSV	T-VEC (37)	TR (ORR: 40.5%)	NA	IT injection at an initial dose of 10^6^ PFU/mL (up to 4 mL total). 3 weeks after, at a dose of 10^8^ PFU/mL (up to 4 mL total) q2w for up to 8 doses over a 15-week period	IIIC/IV
Andtbacka RHI (2016) [37]	III	Arm1: 70(61–79) Arm2: 66(58–75)	HSV	Arm1: T-VEC (61) Arm2: GM-CSF (26)	TR (ORR: 47.5% v 11.5%), OS	NCT00769704^†^	Refer to OPTiM	IIIB: 9(15%) v 5(19%) IIIC: 17(28%) v 6(23%) IVM1a: 11(18%) v 6(23%) IVM1b: 15(25%) v 4(15%) IVM1c: 9(15%) v 5(19%)
Andtbacka RHI (2016) [49]	III	63.07 ± 13.68	HSV	T-VEC (277)	TR^‡^ (ORR: 32.5%, 17.5%, 13.9%), OS	NCT00769704^†‡^	Refer to OPTiM	IIIB: 22(8%) IIIC: 66(22%) IVM1a: 75(25%) IVM1b: 64(22%) IVM1c: 67(23%)
Bramante S (2015) [32]	NA	44(38–74)	Adenovirus	Ad5/3-D24-GM-CSF (9)	TR (DCR: 44.4%), OS	NA	IT (or a combination of IT and IV) injections of Ad5/3-D24-GMCSF as a single treatment or serial treatment consisting of three virus administrations within 10 weeks	NA
NCT00289016 (2015) [23]	II	63(15.2)	HSV	T-VEC (50)	TR (ORR: 26%), OS,AE	NCT00289016 (protocol: NCT0257426)	IT injection at an initial dose of 10^6^PFU/mL up to 4 mL (2 mL per tumor). 3 weeks later at 10^8^PFU/mL, q2w for up to 15 weeks	IIIC: 13(26%) IVM1a: 13(26%) IVM1b: 5(10%) IVM1c: 19(38%)
NCT01368276 (2015) [24]	NA	Arm1: 64.2 ± 13.2 Arm2: 64.2 ± 13.2	HSV	Arm1: T-VEC (28) Arm2: GM-CSF (3)	TR (ORR: 57.1% v 100%), AE	NCT01368276^§^	Arm1: IT injection at a dose of 10^8^ PFU/mL on Days 1 and 15 of each 28-day cycle for up to 12 months or until a complete response Arm2: administered at a dose of 125 μg/m^2^/day subcutaneously for 14 consecutive days followed by 14 days of rest, in 28-day treatment cycles for up to 12 months or until a complete response	IIB: 1(3.2%) IIIC: 6(19.4%) IVM1a: 10(32.3%) IVM1b: 9(29.0%) IVM1c: 5(16.1%)
NCT00651157 (2014) [25]	NA	65(22–80)	Reovirus	REOLYSIN® (23)	TR (ORR: 0%), AE	NCT00651157	IV injection over 60 min on days 1–5. Repeats every 28 days for up to 12 courses in the absence of disease progression or unacceptable toxicity	IV: 23(100%)
Galanis E (2012) [44]	II	65(22–80)	Reovirus	REOLYSIN® (21)	TR (DCR: 28.6%; ORR: 0%), OS, PFS, AE	NCT00984464^¶^	IV injection at a dose of 3 × 10^10^ TCID_50_ per day over 60 min daily on days 1–5 of a 4-week cycle	M1a: 3 (14.3%) M1b: 2 (9.5%) M1c: 16 (76.2%)
Hwang T (2011) [14]	NA	65.1(46–85)	Poxvirus	JX-594 (8)	TR (DCR: 37.5%; ORR: 0%), AE	NA	IT injection every week for a target total of six injections over 6 weeks (range 2–9 injections)	Mx: 1 (10%) M1b: 2 (20%) M1c: 7 (70%)
Senzer NN (2009) [33]	II	62(34–88)	HSV	JS1/34.5-/47-/GM-CSF (50)	TR (DCR: 50%; ORR: 26%), OS, PFS, AE	NA	IT injection of up to 4 mL at 10^6^ PFU/mL, followed 3 weeks later by injections of up to 4 mL at 10^8^ PFU/mL repeated every 2 weeks	IIIc: 10(20%) IVM1a: 16(32%) IVM1b: 4(8%) IVM1c: 20(40%)
Kaufman HL (2005) [45]	I	54(34–74)	Poxvirus	rV-B7.1 (11)	TR (DCR: 27.3%; ORR: 9.1%), AE	NCT00004148^¶^	monthly intralesional injections, and each cycle consisted of 3 vaccine doses. Two vaccine concentrations, 4.26 × 10^7^ and 4.26 × 10^8^PFU	IVM1a: 4(33.3%) IVM1b: 3(25%) IVM1c: 5(41.7%)
Zajac P (2003) [50]	I//II	62 ± 15	Poxvirus	rVV-mel-B7 (18)	TR (ORR: 0%), AE	NCT00116597^¶^	Two cycles: on day 3 of the first week, a 10^7^ PFU dose of virus was administered intradermally in the abdominal region. The second cycle (from day 57 to day 99) was a higher dose of virus 10^8^ PFU	III: 5(25%) IV: 15(75%)

*HSV* Herpes simplex virus, *T-VEC* Talimogene laherparepvec, *CVA21* Coxsackievirus A21, *GM-CSF* Granulocyte macrophage colony-stimulating factor, *NA* Not available. *TR* Treatment response, *CR* Complete response, *PD* Progression disease, *DCR* Disease control rate, *ORR* Overall response rate, *OS* Overall survival, *PFS* Progression-free survival, *AE* Adverse events, *PFU* Plaque forming units, *IT* Intratumoral/intralesional, *IV* Intravenous

^†^Conclusion was reported with different sample size during the clinical trial

^‡^Evaluation of patients with injected lesions, non-visceral and visceral lesions

^§^An extension protocol in study NCT00769704

^¶^No result posted on ClinicalTrials.org

### Efficacy

#### Changes in response to virotherapies

**Fig. 1:**
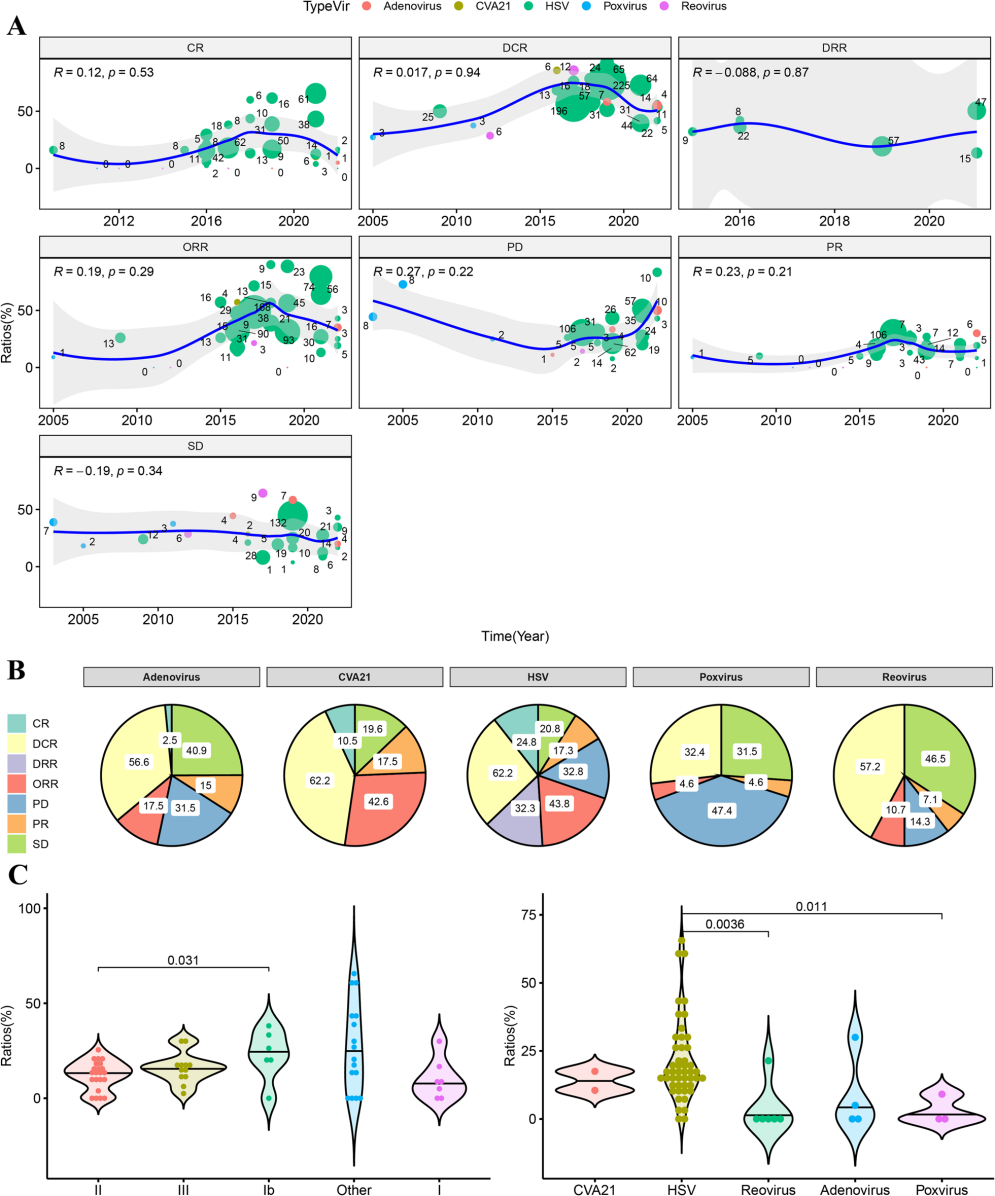
The comparison of therapeutic efficacy with oncolytic virotherapy in the treatment of advance melanoma in recent decades. **A** Changes of treatment response over time 2000 to 2022. The size of dots was weighted by the number of events. The local regression fitting line with 95% confidence region was drawn according to the changes of time (x) and ratio (y). **B** Comparison of the average treatment response in different oncolytic virotherapy. **C** Wilcoxon-test of changes in ORR grouped by clinical trials phase (left) and oncolytic virotherapy (right). (*HSV* Herpes simplex virus, *CVA21* Coxsackievirus A21, *CR* Complete response, *PR* Partial response, *SD* Stable disease, *PD* Progressive disease, *DCR* Disease control rate, *DRR* Durable response rate, *ORR* Overall/objective response rate, *AE* Adverse effect, DCR = CR+PR+SD, ORR = CR+PR. Notes in Fig. 2B: Due to multiple studies have different observations and events. The sum of the proportions in a single pie chart does not equal to 100%.)

Correlation test results showed that there were no significant changes in CR, PR, SD, PD, ORR, DCR or DRR from 2003 to 2022 (Fig. 1A). Meanwhile, their *R* values were all less than 0.3. Interestingly, the mean DCR values for virotherapies CVA21 and HSV were the same (62.2%), and the ORR of HSV was slightly greater than that of CVA21 (43.8% vs 42.6%) (Fig. 1B). Among the five kinds of oncolytic viruses, HSV had the highest mean CR (24.8%). For the endpoint of PD, the mean value of virotherapy for poxvirus was the highest (47.4%), followed by adenovirus (31.5%). In addition, the Wilcoxon-test was performed for difference in CR+PR (Fig. 1C) according to the phase and virotherapy of clinical trials. Significant results were found in phase II versus Ib (P = 0.031), HSV versus reovirus (P = 0.0036) and HSV versus poxvirus (P = 0.011). However, no significant difference was found when the same test method was performed for CR+PR+SD.

In the fixed model of different virotherapies (Additional file 2: Table S1), the ORR of HSV (IV = 0.18, 95% CI [0.16; 0.19]) and CVA21 (IV = 0.19, 95% CI [0.10; 0.30]) were nearly 3-, 9-, and 18-fold that of adenovirus (IV = 0.06; 95% CI [0.00; 0.22]), poxvirus (IV = 0.02; 95% CI [0.00; 0.14]) and reovirus (IV = 0.01; 95% CI [0.00; 0.04]), respectively.

In the pooled IV of the fixed model of HSV (T-VEC) and HSV+(T-VEC+ICIs) (Additional file 2: Table S2), the ORR of HSV (IV = 0.36, 95% CI [0.34; 0.39]) was lower than that of HSV+(IV = 0.43, 95% CI [0.39; 0.47]), while the CR of HSV (IV = 0.19, 95% CI [0.17; 0.21]) was higher than that of HSV+(IV = 0.15, 95% CI [0.12; 0.18]). There were no significant differences among the paired treatment response groups.

In the pooled IV of fixed model results from five studies [24, 34, 36–38], the two arms compared T-VEC with or without GM-CSF/ICIs to GM-CSF/ICIs monotherapy, suggesting that virotherapy is more efficient in the subgroups CR (RR = 1.80, 95% CI [1.30; 2.51], P < 0.01), ORR (RR = 1.17, 95% CI [1.02; 1.34], P < 0.05), DCR (RR = 1.27, 95% CI [1.15; 1.40], P < 0.01) and DRR (RR = 5.48, 95% CI [2.13; 14.05], P < 0.01) (Additional file 1: Fig. S2, Additional file 2: Table S3).

#### Survival

**Fig. 2:**
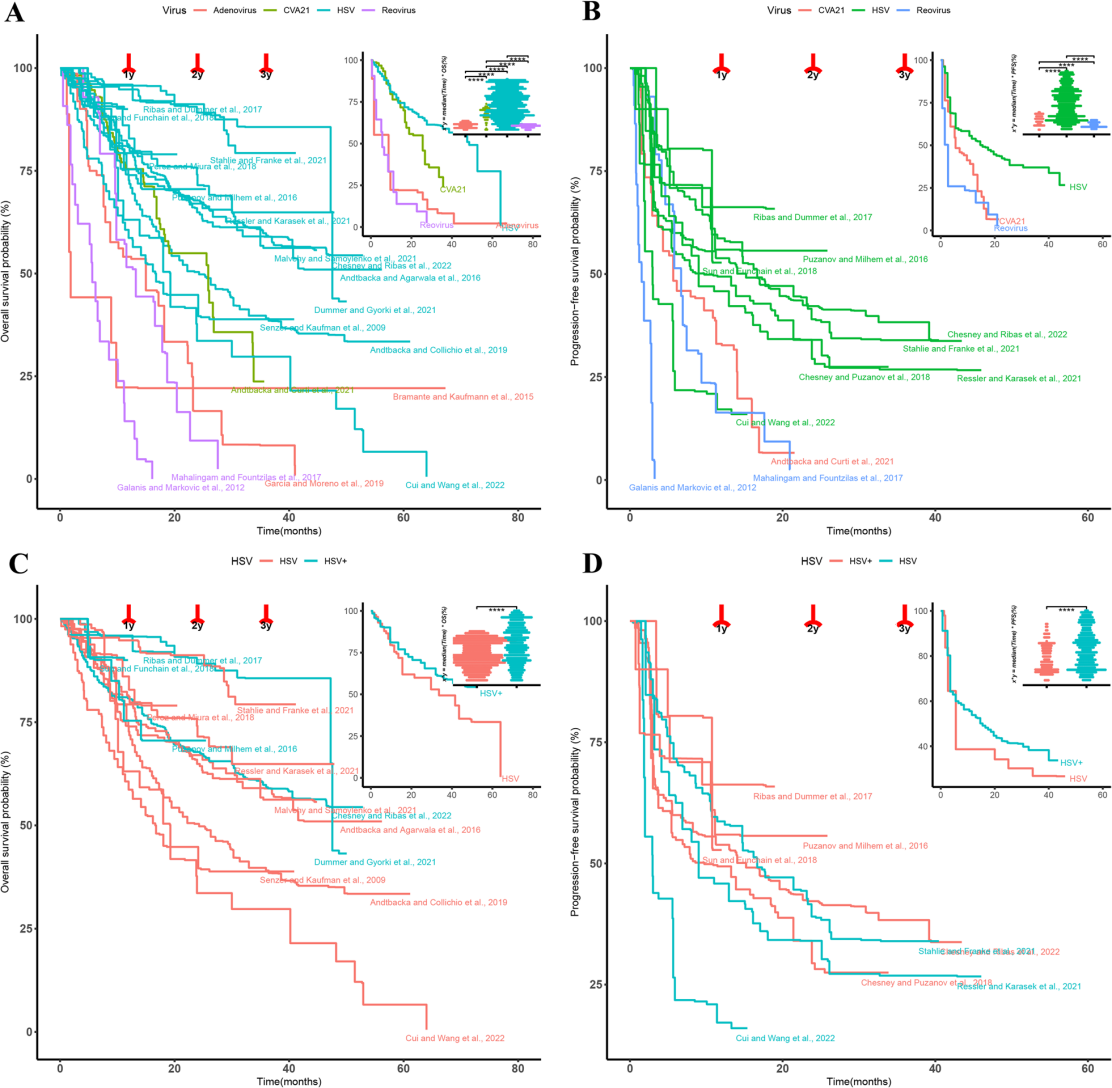
The combined Kaplan–Meier curves of OS or PFS plots. **A**, **B**. The comparison results of combined OS A and PFS B curves of different oncolytic virotherapies in included studies. **C**, **D**. The comparison results of HSV(T-VEC) and HSV + (T-VEC+ICIs) in OS C and PFS D, respectively. The Wilcoxon-test comparison results of 2-year median times were placed in the upper-right corner of each graph

As shown in Fig. 2, in the combined Kaplan–Meier curves of OS or PFS, virotherapy with HSV showed the best therapeutic results. The mean OS at 1-year of HSV, CVA21, reovirus and adenovirus were 6.8 ± 3.6, 6.9 ± 3.4, 6.3 ± 4.0 and 4.8 ± 3.6 months, respectively. The corresponding mean proportions were 88.4 ± 9.2, 88.2 ± 8.0, 61.5 ± 26.7 and 65.1 ± 23.2. The mean OS at 2-year of HSV, CVA21, reovirus and adenovirus were 11.7 ± 6.8, 10.8 ± 6.2, 9.6 ± 6.4 and 9.2 ± 7.4 months, respectively. The corresponding mean proportions of them were 81.6 ± 14.2, 79.4 ± 14.4, 50.3 ± 29.7 and 55.2 ± 25.1, respectively. There was a significant difference (P < 0.001) in the results of the Wilcoxon-test between every two groups of them (top-right of Fig. 2A).

The mean PFS at 1-year of HSV, CVA21 and reovirus were 5.6 ± 3.5, 6.0 ± 3.9 and 3.9 ± 3.4 months, respectively. The corresponding mean proportions of them were 69.8 ± 17.8, 58.5 ± 21.6 and 54.5 ± 28.1, respectively. The mean PFS at 2-year of HSV, CVA21 and reovirus were 9.9 ± 6.9, 9.6 ± 6.2 and 6.1 ± 6.1 months, respectively. The corresponding mean proportions of them were 62.3 ± 18.8, 43.5 ± 28.1 and 47.7 ± 30.5, respectively. There was a significant difference (P < 0.001) in the results of the Wilcoxon-test between every two groups of them (top-right of Fig. 2B).

In studies treated with T-VEC + ICIs (Fig. 2C, HSV +), the mean OS at 1-year was 6.9 ± 3.7 months and was not significantly longer than the 6.8 ± 3.6 months with T-VEC alone (P = 0.11). The mean OS at 2-year was 12.1 ± 6.9 months and was significantly longer than the 11.5 ± 6.8 months with T-VEC alone (P < 0.001). Similarly, there was no significant difference in PFS between HSV+ and HSV at 1-year (P = 0.09), while there was a significant difference in PFS at 2-year (P = 0.006) (Fig. 2D).

### Safety

**Fig. 3:**
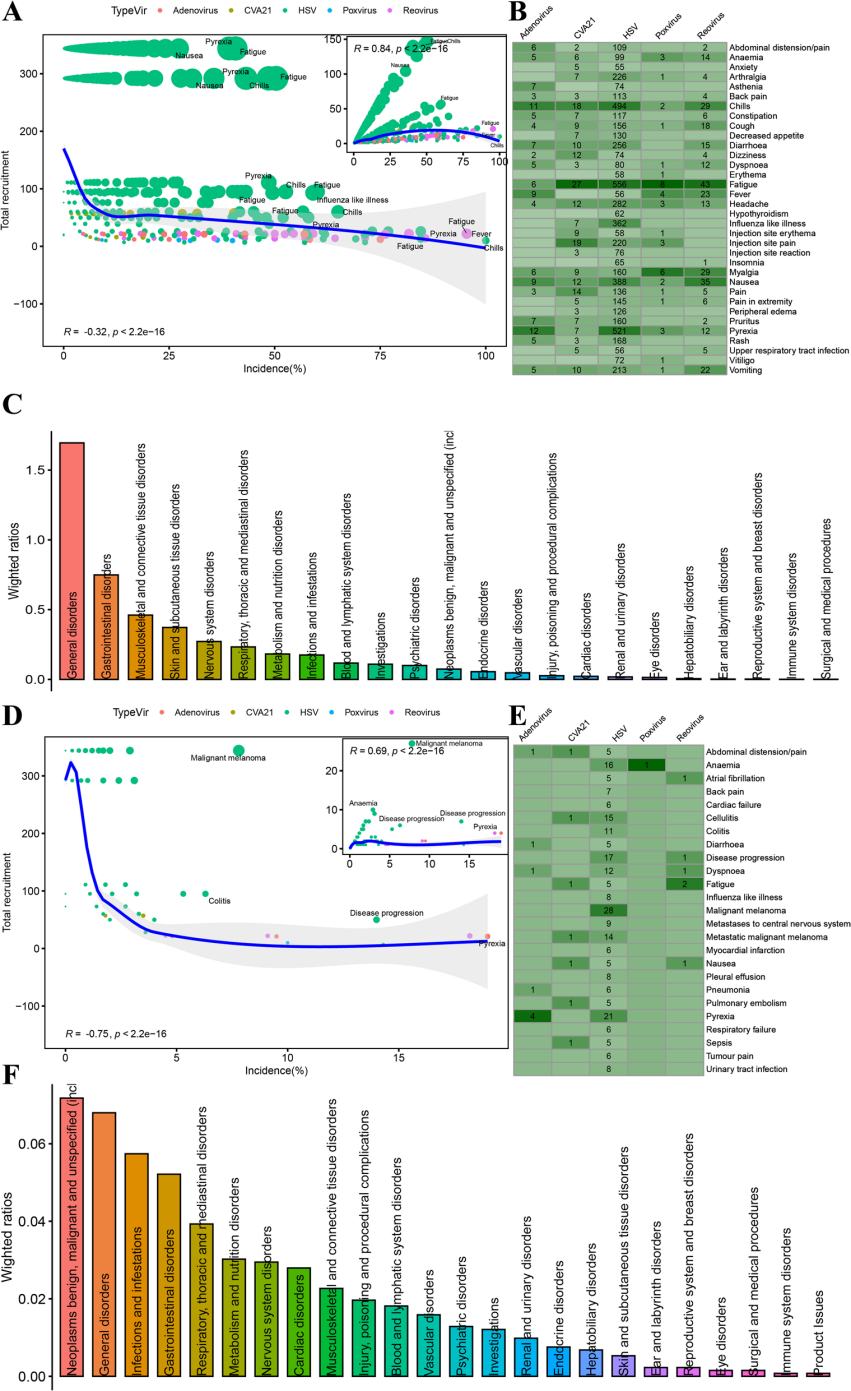
Incidence and frequency of (serious) adverse events of virotherapies. **A**, **D**. Trends in the incidence of common **A** or serious **D **adverse events. The size of dots was weighted by the square root of number of events. The local regression fitting line with 95% confidence region in the main scatter plot was drawn according to the changes of incidence (x) and total recruitment (y), while in the subplot (upper-right) was drawn according to the changes of incidence (x) and total events (y). **B**, **E**. Distribution of frequently common (**B**) or serious (**E**) adverse events in the five kinds of virotherapies. The complete distribution results were attached to supplementary figures S3 and S6, respectively. **C**, **F**. Weighted ratios of common (**C**) or serious (**F**) adverse events in different organ-system

In addition to the difference in efficacy of different virotherapies, AEs are one of the major factors leading to treatment ineffectiveness. Although AEs frequently occur in virotherapy, their incidence is reduced in a large cohort of enrolled patients (Fig. 3A, R = − 0.32, P < 0.001). The heatmap in Fig. 3B shows the most common AE frequencies for each virotherapy. The top six most common AEs were fatigue, chills, nausea, diarrhoea, headache and myalgia. All types of AEs were detailed in supplementary Figure S3 (Additional file 1). For the top six AEs in the T-VEC group compared with the control group (Additional file 1: Fig. S4, Additional file 2: Table S4), the RR of fatigue was 1.44 (95% CI [1.24; 1.66], P < 0.01), the RR of chills was 5.78 (95% CI [4.06; 8.23], P < 0.01), the RR of nausea was 1.59 (95% CI [1.30; 1.94], P < 0.01), the RR of headache was 1.57 (95% CI [1.22; 2.02], P < 0.01) and the RR of myalgia was 2.48 (95% CI [1.62; 3.81], P < 0.01). As shown in Fig. 3C, most AEs occurred in general disorders, gastrointestinal disorders, and musculoskeletal and connective tissue disorders.

However, there was no significant difference between the T-VEC and control groups in serious adverse events (SAEs), such as anaemia, pyrexia, sepsis, dyspnoea, pneumonia and abdominal distension/pain (Additional file 1: Figure S5, Additional file 2: Table S4). In addition, the incidence of SAEs decreased dramatically as the number of enrolled patients increased (Fig. 3D, R = − 0.75, P < 0.01). The heatmap in Fig. 3E shows the most common SAE frequencies for each virotherapy. Most SAEs occurred in neoplasms, general disorders, and infections and infestations (Fig. 3F). All types of SAEs were detailed in supplementary Figure S6 (Additional file 1).

### Efficacy and safety

**Fig. 4:**
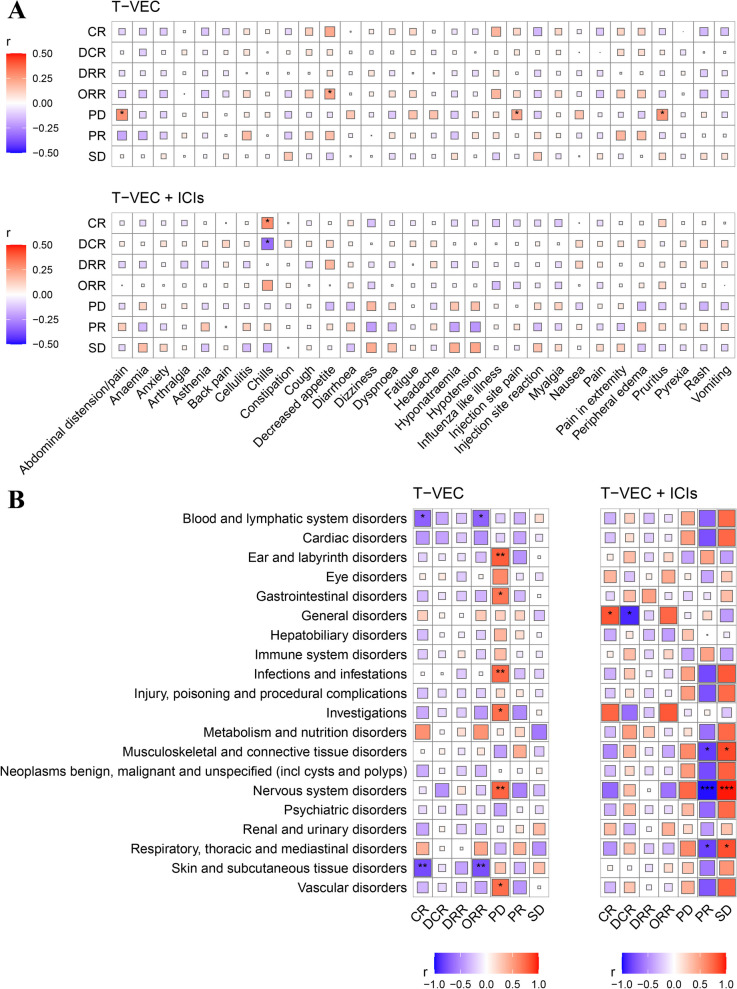
Association between treatment responses and adverse events (AEs). **A**. Correlation test between treatment responses and 30 common AEs for treatments T-VEC and T-VEC+ICIs. **B**. Correlation test between treatment responses and organ-system classification of AEs for treatments T-VEC and T-VEC+ICIs. The positive and negative correlation coefficients are represented by red and blue saturated colors, respectively. The size of the square responds to the strength of correlation between 0 and 1. (*DRR* Durable response rate, *SD* Stable disease. *CR* Complete response, *PR* Partial response, *PD* Progression disease, *DCR* Disease control rate, *ORR* Overall response rate, *ICIs* Immune checkpoint inhibitors. * < 0.05, ** < 0.01, *** < 0.001)

The association between efficacy and safety of T-VEC monotherapy or T-VEC+ICIs was showed in Fig. 4A. In T-VEC monotherapy, abdominal distension/pain, injection site pain and pruritus were significantly positively correlated with PD, and decreased appetite was significantly positively correlated with ORR. In T-VEC+ICIs therapy, chill was significantly positively correlated with CR but negatively correlated with DCR.

In the organ-system classification of AEs treated with T-VEC (Fig. 4B), blood and lymphatic system disorders, skin and subcutaneous tissue disorders were significantly negatively correlated with CR and ORR. Gastrointestinal disorders, general disorders, infections and infestations, nervous system disorders, vascular disorders, ear and labyrinth disorders, and investigations were significantly positively correlated with PD. In treatment with T-VEC+ICIs, respiratory, thoracic and mediastinal disorders, musculoskeletal and connective tissue disorders, and nervous system disorders were significantly positively correlated with SD but were significantly negatively correlated with PR. General disorders were significantly positively correlated with CR and negatively correlated with DCR.

## Discussion

In this study, 34 studies including 2,710 records were used for the first time to evaluate the efficacy and safety of five oncolytic viruses in the treatment of advanced melanoma. Patients treated with HSV and CVA21 showed similar high efficacy in terms of DCR and ORR, while other virotherapies showed poorer outcomes. Compared with other virotherapies, HSV treatment significantly extended OS and PFS. In particular, patients treated with T-VEC+ICIs had significantly longer OS and PFS than those treated with T-VEC monotherapy. Therefore, encoding the blockade function of ICIs into OVs will be more effective than combination therapy, which has achieved significant efficacy in preclinical models [51, 52].

Cancer therapy has entered a new era since the discovery of the oncolytic potential of viruses such as T-VEC, a human herpes simplex virus type 1, which is modified to selectively replicate and secrete human granulocyte–macrophage colony-stimulating factor (GM-CSF) in tumor cells, resulting in activation of the immune response and killing of the infected target [53]. This property limits antitumour effects within the tumor microenvironment and avoids ineffective treatment due to drug resistance to ICIs and the restrictiveness of gene mutations [2, 54]. For example, in an open-label, multicentre study of patients with BRAF mutated metastatic melanoma treated with vemurafenib, only 90 (3.3%) patients endured CR, and 829 (30.6%) patients endured PR [55]. While in a randomized study of advanced melanoma patients treated with T-VEC, patients with mutant BRAF occurred CR in 5 (10.9%) and PR in 9 (19.6%) patients, and patients with wild-type BRAF occurred CR in 5 (11.1%) and PR in 9 (20.0%) patients [36]. In another phase II, open-label, multicentre study of T-VEC in patients with stage IIIB-IVM1c melanoma, the ORR of mutant BRAF was 10 (25%) patients, and that of wild-type BRAF was 20 (29%) patients [9].

Treatment response is a double-edged sword. To some extent, a high treatment response corresponds to more AEs, especially high-dose-dependent AEs, which may lead to cancer treatment termination and failure. In this study, treatment-related AEs such as nausea, fatigue and myalgia were more common in patients treated with T-VEC (+ ICIs) than GM-CSF/ICIs. In the randomized phase III trial OPTiM [36] included in this study, the ORR of T-VEC monotherapy (31.5%) was higher than that of GM-CSF (6.4%) alone, and AEs occurred in 11.3% of patients treated with T-VEC and 4.7% of patients treated with GM-CSF. In another randomized, double-blind phase III trial, MASTERKEY-265 [35], the ORR of combination therapy T-VEC+ pembrolizumab (48.6%) was higher than that of placebo+pembrolizumab (41.3%). Meanwhile, the incidence of grade > 3 treatment-related AEs was 20.3% and 15.7%, respectively, and the incidence of fatal AEs was 13.1% and 12.2%, respectively. Furthermore, no dose-limiting toxicity of T-VEC has been reported in advanced melanoma. The safety and tolerability of T-VEC make it a good treatment option for patients with advanced or unresectable melanoma and those unable to tolerate other treatment toxicities.

Prolonging (progression-free) survival is the primary goal of treatment for patients with advanced melanoma. In current study, T-VEC with or without ICIs had a significantly longer OS at 2-year than other types of OVs, and T-VEC+ICIs was superior to T-VEC alone. This superior outcome was also observed in 2-year PFS for T-VEC+ICIs compared to other OVs, but there was no significant superiority to T-VEC alone at 1-year. Interestingly, in two randomized phase III trials, OPTiM [36] and MASTERKEY-265 [35], no significant difference in OS/PFS was found in T-VEC versus GM-CSF and T-VEC+pembrolizumab versus placebo+pembrolizumab. In the OS of the OPTiM study, T-VEC was found to be superior to GM-CSF only after the removal of 18 patients who had not received allocated therapy, and patients treated with T-VEC as first-line therapy were superior to those treated with T-VEC as second-line therapy. In the staging subsets, T-VEC was superior to GM-CSF in patients with IIIB-IVM1a but not in patients with IVMIb/c.

Although virotherapy appears to be more effective in treating advanced melanoma, there are still some factors that need to be considered before a leap in efficacy can be made. Differences in viral injection volume and the time to peak may result in a delay in the initial response, making it difficult to distinguish false responses from true responses and to decide whether to continue treatment [56]. Furthermore, there is evidence that truly responsive tumors have a “mimicry” of presence with persistent pigmentation, which is in fact an inflammatory infiltrate enriched in melanophages, with no viable tumor cells remaining [57]. This will result in melanoma-specific markers and tissue staining that make it difficult to distinguish between true and false tumor tissue, adversely affecting the manipulation and injection of technicians and affecting viral transmission and optimal tumor perfusion [58]. The host's ability to defend against the virus and the presence of antibodies that neutralize the virus are potential mechanisms of resistance and a robust immune response elicited by the virus that may clear the virus, thereby limiting the activity of virotherapy [59].

The limitation of this study is that injection methods (such as intratumoral and intravenous) may contribute to the heterogeneity in efficacy assessment. The lack of data on patients who withdrew or changed treatment due to AEs may have influenced the results of this study. More studies of other rarely used virotherapies (such as CVA21) need to be included in future updated assessments.

## Conclusion

Considering the relative safety and tolerability of OVs and the absence of reports of dose-limiting-dependent toxicities, more patients treated with T-VEC with or without ICIs should be added to future assessment analyses. There is still a long way to go before it can be used as a first-line therapy for patients with advanced or unresectable melanoma.

### Supplementary Information

**Additional file 1.** Supplementary Figures S1-S6.

**Additional file 2.** Supplementary Tables S1-S4.

**Additional file 3.** Quality assessment results of included studies.

## Data Availability

The data sets that support the conclusions of this article are included within the article and the included publications and registered trials.

## Abbreviations

HSV: Herpes simplex virus
CVA21: Coxsackievirus A21
CR: Complete response
PR: Partial response
SD: Stable disease
PD: Progressive disease
DCR: Disease control rate
DRR: Durable response rate
ORR: Overall/objective response rate
AE: Adverse effect
DCR: CR+PR+SD
ORR: CR+PR
T-VEC: Talimogene laherparepvec
GM-CSF: Granulocyte macrophage colony-stimulating factor
OS: Overall survival
PFS: Progression-free survival
AE: Adverse events
PFU: Plaque forming units
