# Supplementary material for: The efficacy and safety assessment of oncolytic virotherapies in the treatment of advanced melanoma: a systematic review and meta-analysis
Source: Virol J. 2023 Nov 2;20:252. doi: 10.1186/s12985-023-02220-x (PMC10623758; doi:10.1186/s12985-023-02220-x)
Supplement: Supplementary file 1 — Additional file 1. Supplementary Figures S1-S6. [file 12985_2023_2220_MOESM1_ESM.pdf]

Supplementary figure legends:

Figure S1. Flow diagram for databases and registers identification and screening.

Figure S2. Comparison of the pooled risk ratio of treatment response with or without T-VEC.

Figure S3. Detailed frequency of all types of common adverse events.

Figure S4. Comparison of the pooled risk ratio of top six common adverse events in treatments with or without T-VEC.

Figure S5. Comparison of the pooled risk ratio of top six serious adverse events in treatments with or without T-VEC.

Figure S6. Detailed frequency of all types of serious adverse events.

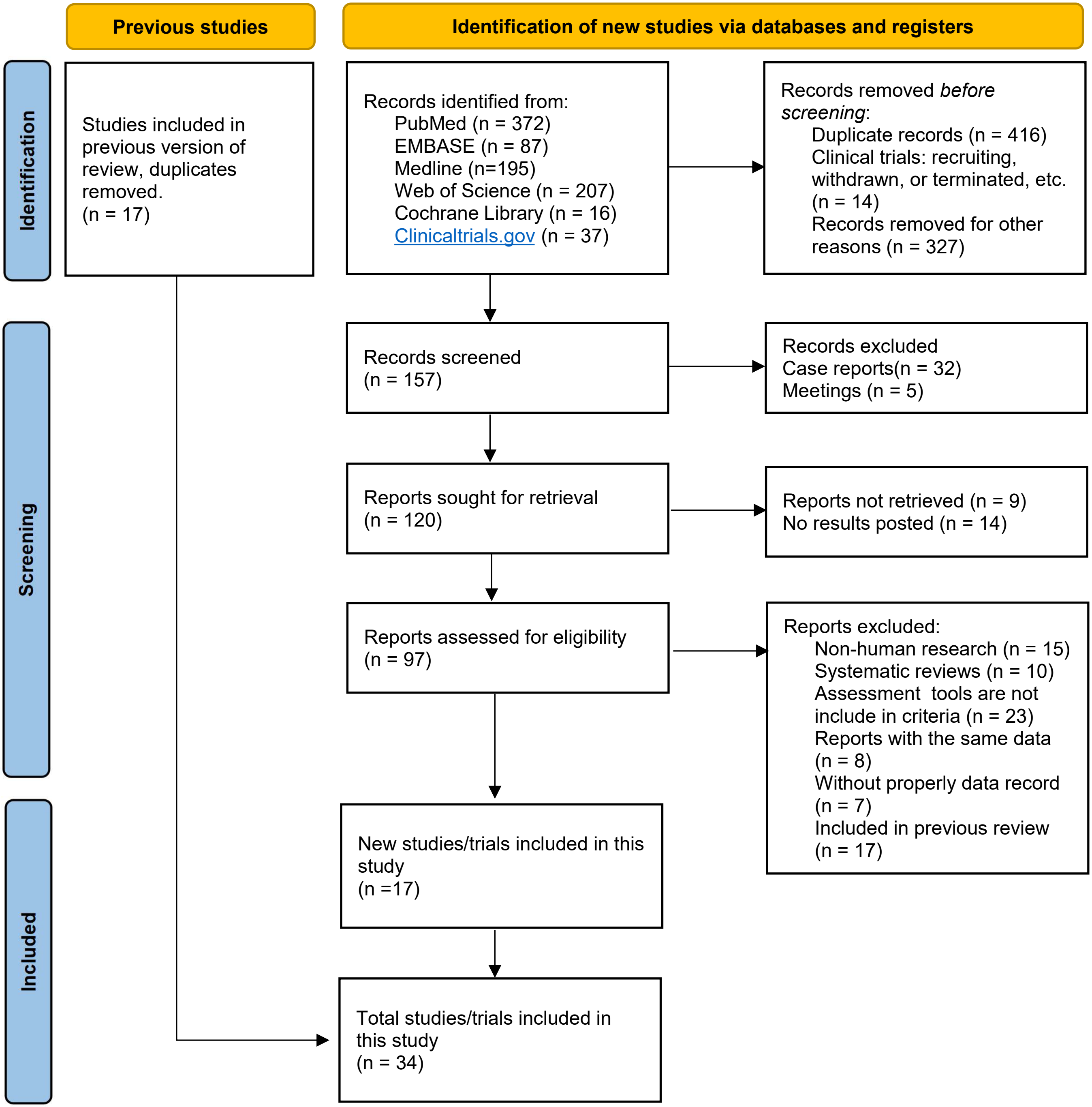

Previous studies
Identification of new studies via databases and registers
Identification
Studies included in previous version of review, duplicates removed. (n = 17)
Records identified from: PubMed (n = 372) EMBASE (n = 87) Medline (n=195) Web of Science (n = 207) Cochrane Library (n = 16) Clinicaltrials.gov (n = 37)
Records removed *before screening*: Duplicate records (n = 416) Clinical trials: recruiting, withdrawn, or terminated, etc. (n = 14) Records removed for other reasons (n = 327)
Screening
Records screened (n = 157)
Records excluded Case reports(n = 32) Meetings (n = 5)
Reports sought for retrieval (n = 120)
Reports not retrieved (n = 9) No results posted (n = 14)
Reports assessed for eligibility (n = 97)
Reports excluded: Non-human research (n = 15) Systematic reviews (n = 10) Assessment tools are not include in criteria (n = 23) Reports with the same data (n = 8) Without properly data record (n = 7) Included in previous review (n = 17)
Included
New studies/trials included in this study (n =17)
Total studies/trials included in this study (n = 34)

Study or Subgroup	Experimental Events	Experimental Total	Control Events	Control Total	Weight (common)	Weight (random)	Risk Ratio IV, Fixed + Random, 95% CI
**Group = DRR**							
Andtbacka RHI_2019	57	295	2	141	0.2%	2.2%	13.62 [3.37; 55.00]
Andtbacka RHI_2016	22	61	1	26	0.1%	1.4%	9.38 [1.33; 65.96]
NCT01368276_2015	9	28	1	3	0.1%	1.7%	0.96 [0.18; 5.22]
**Total (fixed effect, 95% CI)**		**384**		**170**	**0.4%**	**--**	**5.48 [2.13; 14.05]**
**Total (random effects, 95% CI)**					**--**	**5.4%**	**5.07 [0.97; 26.42]**
Heterogeneity: $Tau^2 = 1.3987$; $Chi^2 = 5.99$, df = 2 (P = 0.05); $I^2 = 67\%$							
Test fixed effect: Z = 3.54 (P < 0.01)							
Test random effects: Z = 1.93 (P = 0.05)							
**Group = CR**							
Andtbacka RHI_2019	50	295	1	141	0.1%	1.4%	23.90 [3.34; 171.24]
Chesney J_2018	13	98	7	100	0.5%	3.5%	1.90 [0.79; 4.55]
Ribas A_2017	62	346	40	346	2.9%	5.0%	1.55 [1.07; 2.24]
Andtbacka RHI_2016	18	61	1	26	0.1%	1.4%	7.67 [1.08; 54.50]
**Total (fixed effect, 95% CI)**		**800**		**613**	**3.6%**	**--**	**1.80 [1.30; 2.51]**
**Total (random effects, 95% CI)**					**--**	**11.3%**	**3.52 [1.12; 11.05]**
Heterogeneity: $Tau^2 = 0.9395$; $Chi^2 = 9.37$, df = 3 (P = 0.02); $I^2 = 68\%$							
Test fixed effect: Z = 3.50 (P < 0.01)							
Test random effects: Z = 2.15 (P = 0.03)							
**Group = PR**							
Andtbacka RHI_2019	43	295	8	141	0.7%	3.9%	2.57 [1.24; 5.32]
Chesney J_2018	25	98	11	100	0.9%	4.2%	2.32 [1.21; 4.45]
Ribas A_2017	106	346	103	346	7.6%	5.3%	1.03 [0.82; 1.29]
Andtbacka RHI_2016	11	61	2	26	0.2%	2.1%	2.34 [0.56; 9.85]
**Total (fixed effect, 95% CI)**		**800**		**613**	**9.4%**	**--**	**1.22 [0.99; 1.49]**
**Total (random effects, 95% CI)**					**--**	**15.5%**	**1.74 [1.00; 3.02]**
Heterogeneity: $Tau^2 = 0.1930$; $Chi^2 = 10.71$, df = 3 (P = 0.01); $I^2 = 72\%$							
Test fixed effect: Z = 1.89 (P = 0.06)							
Test random effects: Z = 1.96 (P = 0.05)							
**Group = SD**							
Andtbacka RHI_2019	132	295	71	141	9.1%	5.3%	0.89 [0.72; 1.09]
Chesney J_2018	19	98	24	100	1.4%	4.5%	0.81 [0.47; 1.38]
Ribas A_2017	28	346	30	346	1.6%	4.6%	0.93 [0.57; 1.53]
**Total (fixed effect, 95% CI)**		**739**		**587**	**12.1%**	**--**	**0.88 [0.74; 1.06]**
**Total (random effects, 95% CI)**					**--**	**14.5%**	**0.88 [0.74; 1.06]**
Heterogeneity: $Tau^2 = 0$; $Chi^2 = 0.16$, df = 2 (P = 0.92); $I^2 = 0\%$							
Test fixed effect: Z = −1.33 (P = 0.18)							
Test random effects: Z = −1.33 (P = 0.18)							
**Group = PD**							
Andtbacka RHI_2019	62	295	42	141	3.4%	5.1%	0.71 [0.50; 0.99]
Chesney J_2018	31	98	33	100	2.4%	4.9%	0.96 [0.64; 1.43]
Ribas A_2017	106	346	120	346	8.5%	5.3%	0.88 [0.71; 1.09]
**Total (fixed effect, 95% CI)**		**739**		**587**	**14.3%**	**--**	**0.85 [0.72; 1.00]**
**Total (random effects, 95% CI)**					**--**	**15.3%**	**0.85 [0.72; 1.00]**
Heterogeneity: $Tau^2 = 0$; $Chi^2 = 1.64$, df = 2 (P = 0.44); $I^2 = 0\%$							
Test fixed effect: Z = −1.95 (P = 0.05)							
Test random effects: Z = −1.95 (P = 0.05)							
**Group = ORR**							
Andtbacka RHI_2019	93	295	9	141	0.9%	4.2%	4.94 [2.57; 9.50]
Chesney J_2018	38	98	18	100	1.6%	4.7%	2.15 [1.32; 3.50]
Ribas A_2017	168	346	143	346	14.2%	5.4%	1.17 [1.00; 1.39]
Andtbacka RHI_2016	29	61	3	26	0.3%	2.9%	4.12 [1.38; 12.33]
NCT01368276_2015	16	28	3	3	4.0%	5.1%	0.58 [0.42; 0.79]
**Total (fixed effect, 95% CI)**		**828**		**616**	**21.0%**	**--**	**1.17 [1.02; 1.34]**
**Total (random effects, 95% CI)**					**--**	**22.2%**	**1.84 [0.84; 4.03]**
Heterogeneity: $Tau^2 = 0.7087$; $Chi^2 = 49.13$, df = 4 (P < 0.01); $I^2 = 92\%$							
Test fixed effect: Z = 2.25 (P = 0.02)							
Test random effects: Z = 1.52 (P = 0.13)							
**Group = DCR**							
Andtbacka RHI_2019	225	295	80	141	15.7%	5.4%	1.34 [1.15; 1.57]
Chesney J_2018	57	98	32	100	3.6%	5.1%	1.82 [1.30; 2.53]
Ribas A_2017	196	346	173	346	19.9%	5.4%	1.13 [0.98; 1.30]
**Total (fixed effect, 95% CI)**		**739**		**587**	**39.2%**	**--**	**1.27 [1.15; 1.40]**
**Total (random effects, 95% CI)**					**--**	**15.9%**	**1.35 [1.07; 1.72]**
Heterogeneity: $Tau^2 = 0.0334$; $Chi^2 = 7.55$, df = 2 (P = 0.02); $I^2 = 74\%$							
Test fixed effect: Z = 4.64 (P < 0.01)							
Test random effects: Z = 2.48 (P = 0.01)							
**Total (fixed effect, 95% CI)**		**5029**		**3773**	**100.0%**	**--**	**1.14 [1.07; 1.22]**
**Total (random effects, 95% CI)**					**--**	**100.0%**	**1.54 [1.17; 2.01]**
Heterogeneity: $Tau^2 = 0.3427$; $Chi^2 = 127.33$, df = 24 (P < 0.01); $I^2 = 81\%$							

Risk Ratio IV, Fixed + Random, 95% CI

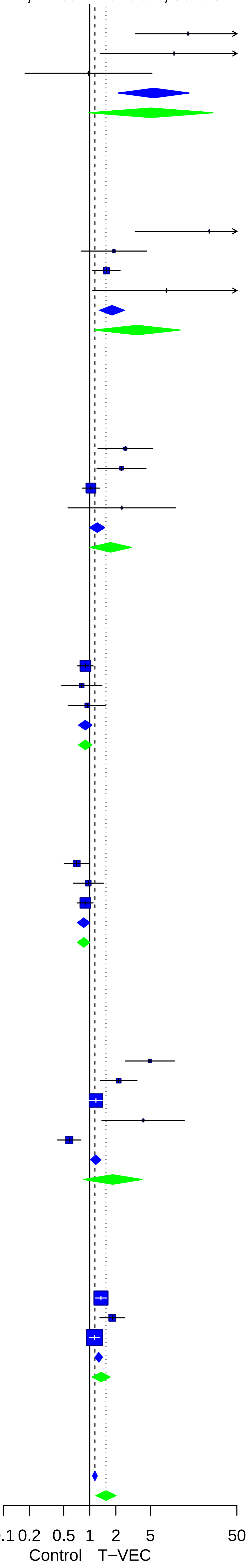

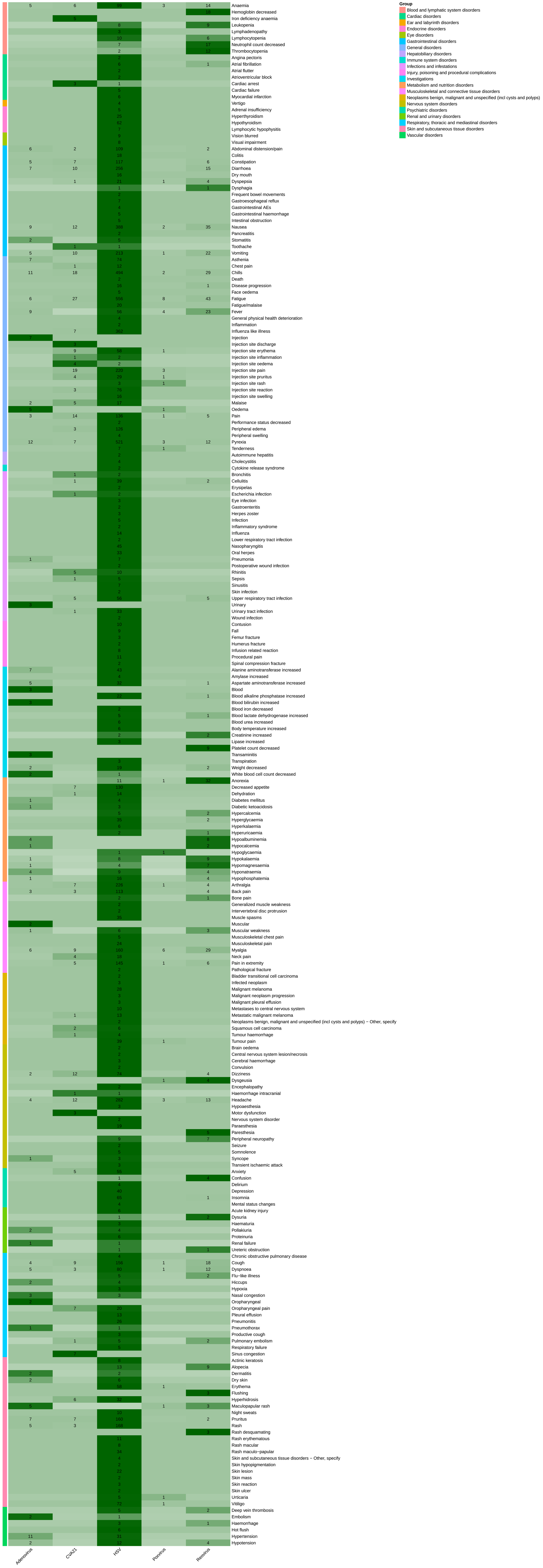
Group
Blood and lymphatic system disorders
Cardiac disorders
Ear and labyrinth disorders
Endocrine disorders
Eye disorders
Gastrointestinal disorders
General disorders
Hepatobiliary disorders
Immune system disorders
Infections and infestations
Injury, poisoning and procedural complications
Investigations
Metabolism and nutrition disorders
Musculoskeletal and connective tissue disorders
Neoplasms benign, malignant and unspecified (incl cysts and polyps)
Nervous system disorders
Psychiatric disorders
Renal and urinary disorders
Respiratory, thoracic and mediastinal disorders
Skin and subcutaneous tissue disorders
Vascular disorders
Adenovirus
CVA21
HSV
Poxvirus
Reovirus
Anaemia
Hemoglobin decreased
Iron deficiency anaemia
Leukopenia
Lymphadenopathy
Lymphocytopenia
Neutrophil count decreased
Thrombocytopenia
Angina pectoris
Atrial fibrillation
Atrial flutter
Atrioventricular block
Cardiac arrest
Cardiac failure
Myocardial infarction
Vertigo
Adrenal insufficiency
Hyperthyroidism
Hypothyroidism
Lymphocytic hypophysitis
Vision blurred
Visual impairment
Abdominal distension/pain
Colitis
Constipation
Diarrhoea
Dry mouth
Dyspepsia
Dysphagia
Frequent bowel movements
Gastroesophageal reflux
Gastrointestinal AEs
Gastrointestinal haemorrhage
Intestinal obstruction
Nausea
Pancreatitis
Stomatitis
Toothache
Vomiting
Asthenia
Chest pain
Chills
Death
Disease progression
Face oedema
Fatigue
Fatigue/malaise
Fever
General physical health deterioration
Inflammation
Influenza like illness
Injection
Injection site discharge
Injection site erythema
Injection site inflammation
Injection site oedema
Injection site pain
Injection site pruritus
Injection site rash
Injection site reaction
Injection site swelling
Malaise
Oedema
Pain
Performance status decreased
Peripheral edema
Peripheral swelling
Pyrexia
Tenderness
Autoimmune hepatitis
Cholecystitis
Cytokine release syndrome
Bronchitis
Cellulitis
Erysipelas
Escherichia infection
Eye infection
Gastroenteritis
Herpes zoster
Infection
Inflammatory syndrome
Influenza
Lower respiratory tract infection
Nasopharyngitis
Oral herpes
Pneumonia
Postoperative wound infection
Rhinitis
Sepsis
Sinusitis
Skin infection
Upper respiratory tract infection
Urinary
Urinary tract infection
Wound infection
Contusion
Fall
Femur fracture
Humerus fracture
Infusion related reaction
Procedural pain
Spinal compression fracture
Alanine aminotransferase increased
Amylase increased
Aspartate aminotransferase increased
Blood
Blood alkaline phosphatase increased
Blood bilirubin increased
Blood iron decreased
Blood lactate dehydrogenase increased
Blood urea increased
Body temperature increased
Creatinine increased
Lipase increased
Platelet count decreased
Transaminitis
Transpiration
Weight decreased
White blood cell count decreased
Anorexia
Decreased appetite
Dehydration
Diabetes mellitus
Diabetic ketoacidosis
Hypercalcemia
Hyperglycaemia
Hyperkalaemia
Hyperuricaemia
Hypoalbuminemia
Hypocalcemia
Hypoglycaemia
Hypokalaemia
Hypomagnesaemia
Hyponatraemia
Hypophosphatemia
Arthralgia
Back pain
Bone pain
Generalized muscle weakness
Intervertebral disc protrusion
Muscle spasms
Muscular
Muscular weakness
Musculoskeletal chest pain
Musculoskeletal pain
Myalgia
Neck pain
Pain in extremity
Pathological fracture
Bladder transitional cell carcinoma
Infected neoplasm
Malignant melanoma
Malignant neoplasm progression
Malignant pleural effusion
Metastases to central nervous system
Metastatic malignant melanoma
Neoplasms benign, malignant and unspecified (incl cysts and polyps) − Other, specify
Squamous cell carcinoma
Tumour haemorrhage
Tumour pain
Brain oedema
Central nervous system lesion/necrosis
Cerebral haemorrhage
Convulsion
Dizziness
Dysgeusia
Encephalopathy
Haemorrhage intracranial
Headache
Hypoaesthesia
Motor dysfunction
Nervous system disorder
Paraesthesia
Paresthesia
Peripheral neuropathy
Seizure
Somnolence
Syncope
Transient ischaemic attack
Anxiety
Confusion
Delirium
Depression
Insomnia
Mental status changes
Acute kidney injury
Dysuria
Haematuria
Pollakiuria
Proteinuria
Renal failure
Ureteric obstruction
Chronic obstructive pulmonary disease
Cough
Dyspnoea
Flu−like illness
Hiccups
Hypoxia
Nasal congestion
Oropharyngeal
Oropharyngeal pain
Pleural effusion
Pneumonitis
Pneumothorax
Productive cough
Pulmonary embolism
Respiratory failure
Sinus congestion
Actinic keratosis
Alopecia
Dermatitis
Dry skin
Erythema
Flushing
Hyperhidrosis
Maculopapular rash
Night sweats
Pruritus
Rash
Rash desquamating
Rash erythematous
Rash macular
Rash maculo−papular
Skin and subcutaneous tissue disorders − Other, specify
Skin hypopigmentation
Skin lesion
Skin mass
Skin reaction
Skin ulcer
Urticaria
Vitiligo
Deep vein thrombosis
Embolism
Haemorrhage
Hot flush
Hypertension
Hypotension

Study or Subgroup	Experimental Events	Experimental Total	Control Events	Control Total	Weight (common)	Weight (random)	Risk Ratio MH, Fixed + Random, 95% CI
**TypeAE = Diarrhoea**							
Andtbacka RHI_2019	55	292	14	127	3.3%	5.3%	1.71 [0.99; 2.96]
Chesney J_2018	41	95	35	95	5.8%	6.3%	1.17 [0.83; 1.66]
Ribas A_2017	74	344	73	344	12.2%	6.6%	1.01 [0.76; 1.35]
**Total (fixed effect, 95% CI)**		**731**		**566**	**21.3%**	**--**	**1.16 [0.95; 1.43]**
**Total (random effects, 95% CI)**					**--**	**18.1%**	**1.16 [0.93; 1.45]**
Heterogeneity: $Tau^2 = 0.0053$; $Chi^2 = 2.76$, df = 2 (P = 0.25); $I^2 = 27\%$							
Test fixed effect: Z = 1.43 (P = 0.15)							
Test random effects: Z = 1.28 (P = 0.20)							
**TypeAE = Nausea**							
Andtbacka RHI_2019	104	292	25	127	5.8%	6.1%	1.81 [1.23; 2.66]
Chesney J_2018	38	95	27	95	4.5%	6.0%	1.41 [0.94; 2.11]
Ribas A_2017	94	344	61	344	10.2%	6.6%	1.54 [1.16; 2.05]
**Total (fixed effect, 95% CI)**		**731**		**566**	**20.5%**	**--**	**1.59 [1.30; 1.94]**
**Total (random effects, 95% CI)**					**--**	**18.7%**	**1.57 [1.29; 1.92]**
Heterogeneity: $Tau^2 = 0$; $Chi^2 = 0.82$, df = 2 (P = 0.66); $I^2 = 0\%$							
Test fixed effect: Z = 4.53 (P < 0.01)							
Test random effects: Z = 4.46 (P < 0.01)							
**TypeAE = Chills**							
Andtbacka RHI_2019	142	292	11	127	2.6%	5.1%	5.61 [3.15; 10.00]
Chesney J_2018	50	95	4	95	0.7%	3.3%	12.50 [4.70; 33.24]
Ribas A_2017	74	344	17	344	2.8%	5.5%	4.35 [2.63; 7.22]
**Total (fixed effect, 95% CI)**		**731**		**566**	**6.1%**	**--**	**5.78 [4.06; 8.23]**
**Total (random effects, 95% CI)**					**--**	**13.9%**	**5.80 [3.65; 9.24]**
Heterogeneity: $Tau^2 = 0.0616$; $Chi^2 = 3.53$, df = 2 (P = 0.17); $I^2 = 43\%$							
Test fixed effect: Z = 9.74 (P < 0.01)							
Test random effects: Z = 7.41 (P < 0.01)							
**TypeAE = Fatigue**							
Andtbacka RHI_2019	147	292	46	127	10.7%	6.7%	1.39 [1.07; 1.80]
Chesney J_2018	57	95	40	95	6.7%	6.6%	1.43 [1.07; 1.90]
Ribas A_2017	140	344	95	344	15.9%	6.8%	1.47 [1.19; 1.82]
**Total (fixed effect, 95% CI)**		**731**		**566**	**33.2%**	**--**	**1.44 [1.24; 1.66]**
**Total (random effects, 95% CI)**					**--**	**20.1%**	**1.44 [1.24; 1.66]**
Heterogeneity: $Tau^2 = 0$; $Chi^2 = 0.12$, df = 2 (P = 0.94); $I^2 = 0\%$							
Test fixed effect: Z = 4.95 (P < 0.01)							
Test random effects: Z = 4.97 (P < 0.01)							
**TypeAE = Myalgia**							
Andtbacka RHI_2019	51	292	7	127	1.6%	4.2%	3.17 [1.48; 6.79]
Chesney J_2018	10	95	4	95	0.7%	2.8%	2.50 [0.81; 7.69]
Ribas A_2017	33	344	16	344	2.7%	5.1%	2.06 [1.16; 3.68]
**Total (fixed effect, 95% CI)**		**731**		**566**	**5.0%**	**--**	**2.48 [1.62; 3.81]**
**Total (random effects, 95% CI)**					**--**	**12.1%**	**2.43 [1.58; 3.71]**
Heterogeneity: $Tau^2 = 0$; $Chi^2 = 0.78$, df = 2 (P = 0.68); $I^2 = 0\%$							
Test fixed effect: Z = 4.17 (P < 0.01)							
Test random effects: Z = 4.07 (P < 0.01)							
**TypeAE = Headache**							
Andtbacka RHI_2019	55	292	12	127	2.8%	5.1%	1.99 [1.11; 3.59]
Chesney J_2018	35	95	22	95	3.7%	5.8%	1.59 [1.01; 2.50]
Ribas A_2017	63	344	45	344	7.5%	6.3%	1.40 [0.98; 1.99]
**Total (fixed effect, 95% CI)**		**731**		**566**	**14.0%**	**--**	**1.57 [1.22; 2.02]**
**Total (random effects, 95% CI)**					**--**	**17.1%**	**1.55 [1.21; 2.00]**
Heterogeneity: $Tau^2 = 0$; $Chi^2 = 1.04$, df = 2 (P = 0.60); $I^2 = 0\%$							
Test fixed effect: Z = 3.49 (P < 0.01)							
Test random effects: Z = 3.44 (P < 0.01)							
**Total (fixed effect, 95% CI)**		**4386**		**3396**	**100.0%**	**--**	**1.74 [1.59; 1.91]**
**Total (random effects, 95% CI)**					**--**	**100.0%**	**1.90 [1.49; 2.42]**
Heterogeneity: $Tau^2 = 0.2104$; $Chi^2 = 71.66$, df = 17 (P < 0.01); $I^2 = 76\%$							
Test for subgroup differences (fixed effect): $Chi^2 = 66.13$, df = 5 (P < 0.01)							
Test for subgroup differences (random effects): $Chi^2 = 42.86$, df = 5 (P < 0.01)							

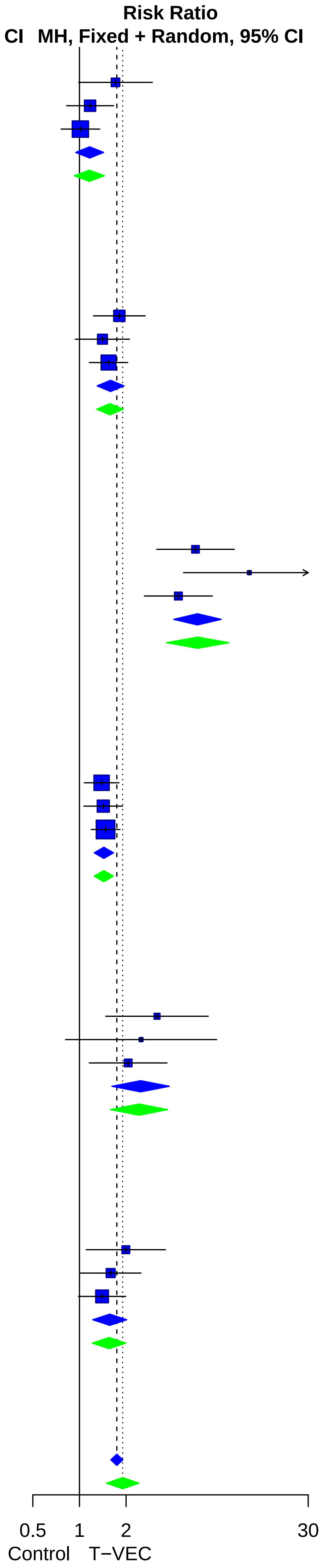

Study or Subgroup	Experimental Events	Experimental Total	Control Events	Control Total	Weight (common)	Weight (random)	Risk Ratio MH, Fixed + Random, 95% CI
**TypeAE = Anaemia**							
Andtbacka RHI_2019	3	292	1	127	4.3%	4.3%	1.30 [0.14; 12.42]
Chesney J_2018	0	95	1	95	4.6%	2.2%	0.33 [0.01; 8.08]
Ribas A_2017	10	344	8	344	24.6%	26.1%	1.25 [0.50; 3.13]
**Total (fixed effect, 95% CI)**		**731**		**566**	**33.5%**	**--**	**1.13 [0.50; 2.54]**
**Total (random effects, 95% CI)**					**--**	**32.6%**	**1.15 [0.51; 2.62]**
Heterogeneity: $Tau^2 = 0$; $Chi^2 = 0.62$, df = 2 (P = 0.73); $I^2 = 0\%$							
Test fixed effect: Z = 0.30 (P = 0.77)							
Test random effects: Z = 0.34 (P = 0.74)							
**TypeAE = Pyrexia**							
Andtbacka RHI_2019	5	292	0	127	2.1%	2.6%	4.79 [0.27; 86.07]
Chesney J_2018	2	95	0	95	1.5%	2.4%	5.00 [0.24; 102.77]
Ribas A_2017	7	344	4	344	12.3%	14.8%	1.75 [0.52; 5.92]
**Total (fixed effect, 95% CI)**		**731**		**566**	**16.0%**	**--**	**2.47 [0.87; 7.01]**
**Total (random effects, 95% CI)**					**--**	**19.9%**	**2.27 [0.79; 6.51]**
Heterogeneity: $Tau^2 = 0$; $Chi^2 = 0.69$, df = 2 (P = 0.71); $I^2 = 0\%$							
Test fixed effect: Z = 1.70 (P = 0.09)							
Test random effects: Z = 1.53 (P = 0.13)							
**TypeAE = Sepsis**							
Andtbacka RHI_2019	1	292	0	127	2.1%	2.2%	1.31 [0.05; 31.88]
Chesney J_2018	1	95	0	95	1.5%	2.2%	3.00 [0.12; 72.72]
Ribas A_2017	1	344	1	344	3.1%	2.9%	1.00 [0.06; 15.92]
**Total (fixed effect, 95% CI)**		**731**		**566**	**6.7%**	**--**	**1.55 [0.28; 8.54]**
**Total (random effects, 95% CI)**					**--**	**7.2%**	**1.51 [0.26; 8.67]**
Heterogeneity: $Tau^2 = 0$; $Chi^2 = 0.27$, df = 2 (P = 0.87); $I^2 = 0\%$							
Test fixed effect: Z = 0.51 (P = 0.61)							
Test random effects: Z = 0.46 (P = 0.65)							
**TypeAE = Dyspnoea**							
Andtbacka RHI_2019	1	292	1	127	4.3%	2.9%	0.43 [0.03; 6.90]
Chesney J_2018	2	95	0	95	1.5%	2.4%	5.00 [0.24; 102.77]
Ribas A_2017	5	344	3	344	9.2%	10.9%	1.67 [0.40; 6.92]
Andtbacka RHI_2016	1	28	0	3	2.7%	2.4%	0.37 [0.02; 7.41]
**Total (fixed effect, 95% CI)**		**759**		**569**	**17.7%**	**--**	**1.47 [0.53; 4.08]**
**Total (random effects, 95% CI)**					**--**	**18.6%**	**1.28 [0.43; 3.80]**
Heterogeneity: $Tau^2 = 0$; $Chi^2 = 2.16$, df = 3 (P = 0.54); $I^2 = 0\%$							
Test fixed effect: Z = 0.73 (P = 0.46)							
Test random effects: Z = 0.44 (P = 0.66)							
**TypeAE = Abdominal distension/pain**							
Chesney J_2018	0	95	0	95	0.0%	0.0%	
Chesney J_2018	1	95	1	95	3.1%	2.9%	1.00 [0.06; 15.76]
Ribas A_2017	2	344	1	344	3.1%	3.8%	2.00 [0.18; 21.95]
Ribas A_2017	1	344	1	344	3.1%	2.9%	1.00 [0.06; 15.92]
**Total (fixed effect, 95% CI)**		**878**		**878**	**9.2%**	**--**	**1.33 [0.30; 5.93]**
**Total (random effects, 95% CI)**					**--**	**9.6%**	**1.32 [0.29; 5.99]**
Heterogeneity: $Tau^2 = 0$; $Chi^2 = 0.19$, df = 2 (P = 0.91); $I^2 = 0\%$							
Test fixed effect: Z = 0.38 (P = 0.71)							
Test random effects: Z = 0.36 (P = 0.72)							
**TypeAE = Pneumonia**							
Chesney J_2018	0	95	1	95	4.6%	2.2%	0.33 [0.01; 8.08]
Ribas A_2017	3	344	4	344	12.3%	9.9%	0.75 [0.17; 3.33]
**Total (fixed effect, 95% CI)**		**439**		**439**	**16.9%**	**--**	**0.64 [0.17; 2.42]**
**Total (random effects, 95% CI)**					**--**	**12.1%**	**0.65 [0.17; 2.50]**
Heterogeneity: $Tau^2 = 0$; $Chi^2 = 0.2$, df = 1 (P = 0.65); $I^2 = 0\%$							
Test fixed effect: Z = −0.66 (P = 0.51)							
Test random effects: Z = −0.63 (P = 0.53)							
**Total (fixed effect, 95% CI)**		**4269**		**3584**	**100.0%**	**--**	**1.37 [0.87; 2.15]**
**Total (random effects, 95% CI)**					**--**	**100.0%**	**1.30 [0.81; 2.07]**
Heterogeneity: $Tau^2 = 0$; $Chi^2 = 6.36$, df = 17 (P = 0.99); $I^2 = 0\%$							
Test for subgroup differences (fixed effect): $Chi^2 = 2.75$, df = 5 (P = 0.74)							
Test for subgroup differences (random effects): $Chi^2 = 2.21$, df = 5 (P = 0.82)							

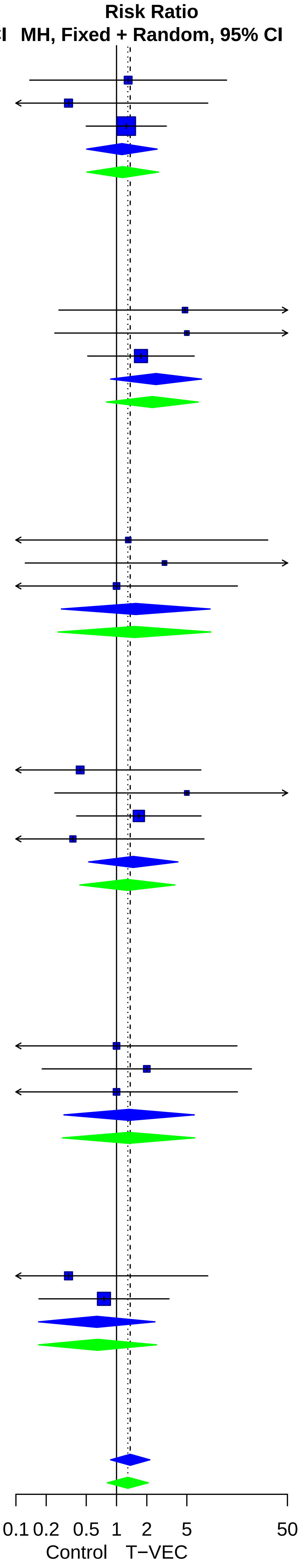

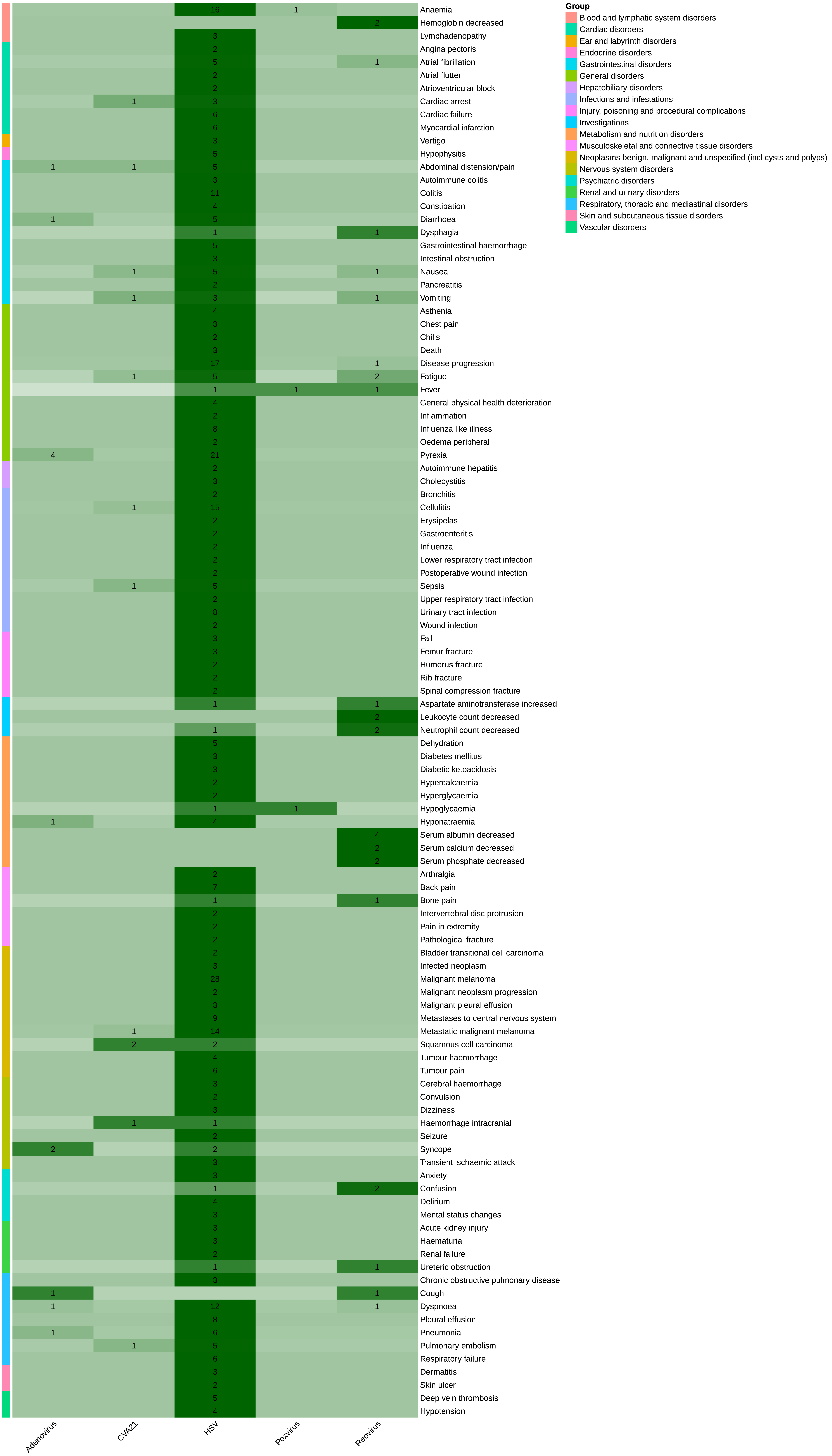
Group
Blood and lymphatic system disorders
Cardiac disorders
Ear and labyrinth disorders
Endocrine disorders
Gastrointestinal disorders
General disorders
Hepatobiliary disorders
Infections and infestations
Injury, poisoning and procedural complications
Investigations
Metabolism and nutrition disorders
Musculoskeletal and connective tissue disorders
Neoplasms benign, malignant and unspecified (incl cysts and polyps)
Nervous system disorders
Psychiatric disorders
Renal and urinary disorders
Respiratory, thoracic and mediastinal disorders
Skin and subcutaneous tissue disorders
Vascular disorders
Anaemia
Hemoglobin decreased
Lymphadenopathy
Angina pectoris
Atrial fibrillation
Atrial flutter
Atrioventricular block
Cardiac arrest
Cardiac failure
Myocardial infarction
Vertigo
Hypophysitis
Abdominal distension/pain
Autoimmune colitis
Colitis
Constipation
Diarrhoea
Dysphagia
Gastrointestinal haemorrhage
Intestinal obstruction
Nausea
Pancreatitis
Vomiting
Asthenia
Chest pain
Chills
Death
Disease progression
Fatigue
Fever
General physical health deterioration
Inflammation
Influenza like illness
Oedema peripheral
Pyrexia
Autoimmune hepatitis
Cholecystitis
Bronchitis
Cellulitis
Erysipelas
Gastroenteritis
Influenza
Lower respiratory tract infection
Postoperative wound infection
Sepsis
Upper respiratory tract infection
Urinary tract infection
Wound infection
Fall
Femur fracture
Humerus fracture
Rib fracture
Spinal compression fracture
Aspartate aminotransferase increased
Leukocyte count decreased
Neutrophil count decreased
Dehydration
Diabetes mellitus
Diabetic ketoacidosis
Hypercalcaemia
Hyperglycaemia
Hypoglycaemia
Hyponatraemia
Serum albumin decreased
Serum calcium decreased
Serum phosphate decreased
Arthralgia
Back pain
Bone pain
Intervertebral disc protrusion
Pain in extremity
Pathological fracture
Bladder transitional cell carcinoma
Infected neoplasm
Malignant melanoma
Malignant neoplasm progression
Malignant pleural effusion
Metastases to central nervous system
Metastatic malignant melanoma
Squamous cell carcinoma
Tumour haemorrhage
Tumour pain
Cerebral haemorrhage
Convulsion
Dizziness
Haemorrhage intracranial
Seizure
Syncope
Transient ischaemic attack
Anxiety
Confusion
Delirium
Mental status changes
Acute kidney injury
Haematuria
Renal failure
Ureteric obstruction
Chronic obstructive pulmonary disease
Cough
Dyspnoea
Pleural effusion
Pneumonia
Pulmonary embolism
Respiratory failure
Dermatitis
Skin ulcer
Deep vein thrombosis
Hypotension
Adenovirus
CVA21
HSV
Poxvirus
Reovirus
