# Supplementary material for: The efficacy and safety assessment of oncolytic virotherapies in the treatment of advanced melanoma: a systematic review and meta-analysis
Source: Virol J. 2023 Nov 2;20:252. doi: 10.1186/s12985-023-02220-x (PMC10623758; doi:10.1186/s12985-023-02220-x)
Supplement: Supplementary file 2 — Additional file 2. Supplementary Tables S1-S4. [file 12985_2023_2220_MOESM2_ESM.pdf]

Table S1. Pooled results of treatment response for different virotherapy

	levels	k	Chi^2	P.val.Q	I^2(%)	Model	IV [LCI; UCI]
ORR/(CR+PR)	Adenovirus	4	9.42	P < 0.05	8	Fixed	0.06[0.00; 0.22]
						Random	0.07[0.01; 0.16]
	CVA21	2	1.97	ns	NA	Fixed	0.19[0.10; 0.30]
						Random	0.19[0.12; 0.27]
	HSV	48	332.06	P < 0.01	82	Fixed	0.18[0.16; 0.19]
						Random	0.19[0.15; 0.23]
	Poxvirus	3	0.96	ns	0	Fixed	0.02[0.00; 0.14]
						Random	0.02[0.00; 0.14]
	Reovirus	6	7.97	ns	0	Fixed	0.01[0.00; 0.04]
						Random	0.01[0.00; 0.05]
DCR/(CR+PR+SD)	Adenovirus	7	25.62	P < 0.01	51	Fixed	0.17[0.10; 0.25]
						Random	0.17[0.03; 0.37]
	CVA21	3	2.47	ns	0	Fixed	0.19[0.10; 0.29]
						Random	0.19[0.12; 0.26]
	HSV	64	492.61	P < 0.01	84	Fixed	0.18[0.17; 0.19]
						Random	0.19[0.16; 0.22]
	Poxvirus	6	11.31	P < 0.05	0	Fixed	0.17[0.08; 0.28]
						Random	0.15[0.03; 0.32]
	Reovirus	8	46.45	P < 0.01	72	Fixed	0.07[0.00; 0.24]
						Random	0.06[0.02; 0.11]

NA: not available. ns: no significance. LCI/UCI: lower/upper confidential interval. IV: inverse-variance. CR: complete response. PR: partial response. SD: stable disease. PD: progressive disease. DCR: disease control rate. ORR: overall/objective response rate. DCR=CR+PR+SD, ORR=CR+PR.

Table S2. Pooled results of treatment arms of T-VEC with or without ICIs.

	levels	k	Chi^2	P.val.Q	I^2(%)	Model	IV[LCI; UCI]
HSV	DRR	5	45.62	P < 0.01	83	Fixed	0.25[0.21; 0.28]
						Random	0.29[0.17; 0.43]
	CR	17	234.54	P < 0.01	91	Fixed	0.19[0.17; 0.21]
						Random	0.22[0.13; 0.32]
	PR	17	13.3	ns	0	Fixed	0.15[0.13; 0.17]
						Random	0.15[0.13; 0.17]
	SD	10	89.84	P < 0.01	84	Fixed	0.26[0.23; 0.29]
						Random	0.20[0.13; 0.29]
	PD	9	69.79	P < 0.01	80	Fixed	0.27[0.24; 0.30]
						Random	0.30[0.18; 0.44]
	ORR	18	231.66	P < 0.01	90	Fixed	0.36[0.34; 0.39]
						Random	0.41[0.31; 0.51]
	DCR	10	84.07	P < 0.01	82	Fixed	0.68[0.64; 0.71]
						Random	0.65[0.53; 0.76]
HSV+	DRR	1	0	NA	NA	Fixed	0.42[0.21; 0.65]
						Random	0.42[0.21; 0.65]
	CR	7	29.65	P < 0.01	59	Fixed	0.15[0.12; 0.18]
						Random	0.20[0.09; 0.35]
	PR	7	23.02	P < 0.01	44	Fixed	0.25[0.21; 0.29]
						Random	0.21[0.13; 0.31]
	SD	6	28.5	P < 0.01	63	Fixed	0.11[0.09; 0.14]
						Random	0.16[0.08; 0.27]
	PD	7	7.75	ns	0	Fixed	0.32[0.28; 0.36]
						Random	0.33[0.26; 0.39]
	ORR	7	56.48	P < 0.01	81	Fixed	0.43[0.39; 0.47]
						Random	0.48[0.29; 0.68]
	DCR	6	11.87	P < 0.05	0	Fixed	0.56[0.52; 0.60]
						Random	0.58[0.48; 0.67]
All virotherapy*	DRR	6	48.28	P < 0.01	80	Fixed	0.25[0.21; 0.28]
						Random	0.31[0.19; 0.44]
	CR	31	302.92	P < 0.01	87	Fixed	0.16[0.10; 0.23]
						Random	0.16[0.15; 0.18]
	PR	32	95.79	P < 0.01	53	Fixed	0.17[0.15; 0.18]
						Random	0.15[0.12; 0.18]
	SD	25	185.52	P < 0.01	82	Fixed	0.23[0.17; 0.30]
						Random	0.20[0.18; 0.22]
	PD	23	98.63	P < 0.01	67	Fixed	0.29[0.27; 0.32]
						Random	0.32[0.25; 0.39]
	ORR	33	351.11	P < 0.01	88	Fixed	0.36[0.28; 0.45]
						Random	0.37[0.35; 0.39]
	DCR	23	136.66	P < 0.01	77	Fixed	0.62[0.60; 0.65]
						Random	0.61[0.53; 0.69]

HSV: Arms with T-VEC. HSV+: Arms with T-VEC plus ipilimumab, nivolumab or pembrolizumab. *Arms with all experimental oncolytic virus. ICIs: immune checkpoint inhibitors. NA: not available. ns: no significance. LCI/UCI: lower/upper confidential interval. IV: inverse-variance. CR: complete response. PR: partial response. SD: stable disease. PD: progressive disease. DCR: disease control rate. DRR: durable response rate. ORR: overall/objective response rate. DCR=CR+PR+SD, ORR=CR+PR.

Table S3. Comparison of the pooled risk ratio of treatment response with or without T-VEC.

levels	k	Chi^2	P.val.Q	I^2(%)	Model	RR[LCI; UCI]	P.val	z.val
DRR	3	5.99	P < 0.05	0	Fixed	5.48[2.13; 14.05]	P < 0.01	3.54
			NA		Random	5.07[0.97; 26.42]	ns	1.93
CR	4	9.37	P < 0.05	7	Fixed	1.80[1.30; 2.51]	P < 0.01	3.5
			NA		Random	3.52[1.12; 11.05]	P < 0.05	2.15
PR	4	10.71	P < 0.05	21	Fixed	1.22[0.99; 1.49]	ns	1.89
			NA		Random	1.74[1.00; 3.02]	ns	1.96
SD	3	0.16	ns	0	Fixed	0.88[0.74; 1.06]	ns	-1.33
			NA		Random	0.88[0.74; 1.06]	ns	-1.33
PD	3	1.64	ns	0	Fixed	0.85[0.72; 1.00]	ns	-1.95
			NA		Random	0.85[0.72; 1.00]	ns	-1.95
ORR	5	49.13	P < 0.01	84	Fixed	1.17[1.02; 1.34]	P < 0.05	2.25
			NA		Random	1.84[0.84; 4.03]	ns	1.52
DCR	3	7.55	P < 0.05	11	Fixed	1.27[1.15; 1.40]	P < 0.01	4.64
			NA		Random	1.35[1.07; 1.72]	P < 0.05	2.48

NA: not available; ns: no significance; LCI/UCI: lower/upper confidential interval; RR: risk ratio. CR: complete response, PR: partial response, SD: stable disease, PD: progressive disease, DCR: disease control rate, DRR: durable response rate, ORR: overall/objective response rate, AE: adverse effect, DCR=CR+PR+SD, ORR=CR+PR.

Table S4. Pooled results of common and serious adverse events

	levels	k	Chi^2	P.val.Q	I^2(%)	Model	IV/RR[LCI; UCI]	P.val	z.val
AE	Diarrhoea	21	78.88	P < 0.01	61	Fixed	0.19[0.17; 0.21]	NA	NA
				NA		Random	0.20[0.15; 0.26]	NA	NA
	Nausea	24	95.06	P < 0.01	64	Fixed	0.29[0.26; 0.31]	NA	NA
				NA		Random	0.30[0.24; 0.37]	NA	NA
	Chills	24	157.55	P < 0.01	79	Fixed	0.37[0.34; 0.40]	NA	NA
				NA		Random	0.41[0.33; 0.49]	NA	NA
	Fatigue	24	234.27	P < 0.01	87	Fixed	0.42[0.39; 0.44]	NA	NA
				NA		Random	0.45[0.34; 0.56]	NA	NA
	Myalgia	21	63.62	P < 0.01	51	Fixed	0.14[0.12; 0.16]	NA	NA
				NA		Random	0.19[0.14; 0.24]	NA	NA
	Headache	21	51.83	P < 0.01	38	Fixed	0.21[0.19; 0.23]	NA	NA
				NA		Random	0.23[0.18; 0.28]	NA	NA
	T-VEC vs. Control								
	Diarrhoea	3	2.76	ns	0	Fixed	1.16[0.95; 1.43]	ns	1.43
				NA		Random	1.16[0.93; 1.45]	ns	1.28
	Nausea	3	0.82	ns	0	Fixed	1.59[1.30; 1.94]	P < 0.01	4.53
				NA		Random	1.57[1.29; 1.92]	P < 0.01	4.46
	Chills	3	3.53	ns	0	Fixed	5.78[4.06; 8.23]	P < 0.01	9.74
				NA		Random	5.80[3.65; 9.24]	P < 0.01	7.41
	Fatigue	3	0.12	ns	0	Fixed	1.44[1.24; 1.66]	P < 0.01	4.95
				NA		Random	1.44[1.24; 1.66]	P < 0.01	4.97
	Myalgia	3	0.78	ns	0	Fixed	2.48[1.62; 3.81]	P < 0.01	4.17
				NA		Random	2.43[1.58; 3.71]	P < 0.01	4.07
	Headache	3	1.04	ns	0	Fixed	1.57[1.22; 2.02]	P < 0.01	3.49
				NA		Random	1.55[1.21; 2.00]	P < 0.01	3.44
SAE	Anaemia	7	11.33	ns	0	Fixed	0.00[0.00; 0.01]	NA	NA
				NA		Random	0.00[0.00; 0.02]	NA	NA
	Pyrexia	8	10.38	ns	0	Fixed	0.02[0.01; 0.03]	NA	NA
				NA		Random	0.02[0.01; 0.03]	NA	NA
	Sepsis	6	7.59	ns	0	Fixed	0.00[0.00; 0.00]	NA	NA
				NA		Random	0.00[0.00; 0.00]	NA	NA
	Dyspnoea	9	11.84	ns	0	Fixed	0.00[0.00; 0.01]	NA	NA
				NA		Random	0.01[0.00; 0.02]	NA	NA
	Abdominal distension/pain	7	6.54	ns	0	Fixed	0.00[0.00; 0.01]	NA	NA
				NA		Random	0.00[0.00; 0.01]	NA	NA
	Pneumonia	6	4.62	ns	0	Fixed	0.01[0.00; 0.01]	NA	NA
				NA		Random	0.01[0.00; 0.01]	NA	NA
	T-VEC vs. Control								
	Anaemia	3	0.62	ns	0	Fixed	1.13[0.50; 2.54]	ns	0.3
				NA		Random	1.15[0.51; 2.62]	ns	0.34
	Pyrexia	3	0.69	ns	0	Fixed	2.47[0.87; 7.01]	ns	1.7
				NA		Random	2.27[0.79; 6.51]	ns	1.53
	Sepsis	3	0.27	ns	0	Fixed	1.55[0.28; 8.54]	ns	0.51
				NA		Random	1.51[0.26; 8.67]	ns	0.46
	Dyspnoea	4	2.16	ns	0	Fixed	1.47[0.53; 4.08]	ns	0.73
				NA		Random	1.28[0.43; 3.80]	ns	0.44
	Abdominal distension/pain	3	0.19	ns	0	Fixed	1.33[0.30; 5.93]	ns	0.38
				NA		Random	1.32[0.29; 5.99]	ns	0.36
	Pneumonia	2	0.2	ns	NA	Fixed	0.64[0.17; 2.42]	ns	-0.66
				NA		Random	0.65[0.17; 2.50]	ns	-0.63

NA: not available. ns: no significance. AE: adverse events. SAE: serious adverse events. IV: inverse-variance. RR: risk ratio.
