# Supplementary material for: The efficacy and safety assessment of oncolytic virotherapies in the treatment of advanced melanoma: a systematic review and meta-analysis
Source: Virol J. 2023 Nov 2;20:252. doi: 10.1186/s12985-023-02220-x (PMC10623758; doi:10.1186/s12985-023-02220-x)
Supplement: Supplementary file 3 — Additional file 3. Quality assessment results of included studies. [file 12985_2023_2220_MOESM3_ESM.pdf]

Cochrane Collaboration's tool for assessing risk of bias in randomized trials

Quality assessment of randomized control trials

Study (Authors, year)	Random sequence generation (selection bias)	Allocation concealment (selection bias)	Blinding of patients and personnel (performance bias)	Blinding of outcome assessment (detection bias complications)	Incomplete outcome data (attrition bias)	Selective reporting (reporting bias)	Other bias
Chesney and Ribas et al., 2022	Low	Low	Low	Low	Low	Low	Low
Andtbacka and Collichio et al., 2019	Low	Low	High	Unclear	Low	Low	Low
Chesney and Puzanov et al., 2018	Low	Low	High	Low	Low	Low	Low
Andtbacka and Agarwala et al., 2016	Low	Low	High	Unclear	Low	Low	Low
NCT01368276, 2015	Low	Low	High	Unclear	Low	Low	Low

Low: Low risk of bias for all key domains. Unclear: Low or unclear risk of bias for all key domains. High: High risk of bias for one or more key domains.

Methodological item for non-randomized studies (MINORS)

Quality assessment of non-randomized studies

Study (Authors, year)	A clearly stated aim	Inclusion of consecutive patients	Prospective collection of data	Endpoints appropriate to the aim of the study	Unbiased assessment of the study endpoint	Follow-up period appropriate to the aim of the study	Loss to follow up less than 5%	Prospective calculation of the study size	Score[$]
Shoushtari and Olszanski et al., 2022	2	2	1	2	2	2	2	2	15
NCT03259425, 2022	2	2	2	2	2	2	2	1	15
Schwarze and Tijtgat et al., 2022	2	2	2	2	2	2	2	1	15
Cui and Wang et al., 2022	2	2	1	2	2	2	2	2	15
Andtbacka and Curti et al., 2021	2	2	2	2	2	2	2	2	16
Dummer and Gyorki et al., 2021[#]	2	2	2	2	2	2	2	2	16
Malvehy and Samoylenko et al., 2021	2	2	2	2	2	2	2	2	16
Ressler and Karasek et al., 2021	2	2	2	2	2	2	2	2	16
Stahlie and Franke et al., 2021	2	2	2	2	2	2	2	2	16
Andtbacka and Amatruda et al., 2019	2	2	2	2	2	2	2	2	16
García and Moreno et al., 2019	2	2	2	2	2	2	1	2	15
Franke and Berger et al., 2019	2	2	1	2	2	2	2	2	15
Louie and Perez et al., 2019	2	2	1	2	2	2	2	2	15
Sun and Funchain et al., 2018	2	2	1	2	2	2	2	2	15
Perez and Miura et al., 2018	2	2	1	2	2	2	2	2	15

Ribas and Dummer et al., 2017	2	2	2	2	2	2	2	2	16
Mahalingam and Fountzilas et al., 2017	2	2	1	2	2	2	2	2	15
Curti and Richards et al., 2016	2	2	2	2	2	2	2	2	16
Puzanov and Milhem et al., 2016	2	2	2	2	2	2	2	2	16
Kaufman and Amatruda et al., 2016	2	2	1	2	2	2	2	2	15
Andtbacka and Ross et al., 2016	2	2	2	2	2	2	2	2	16
Andtbacka and Kaufman et al., 2015	2	2	2	2	2	2	2	2	16
Bramante and Kaufmann et al., 2015	2	2	1	2	2	2	2	2	15
NCT00289016, 2015	2	2	2	2	2	2	2	1	15
NCT00651157, 2014	2	2	2	2	2	2	2	1	15
Galanis and Markovic et al., 2012	2	2	2	2	2	2	2	2	16
Hwang and Moon et al., 2011	2	2	1	2	2	2	2	2	15
Senzer and Kaufman et al., 2009	2	2	1	2	2	2	2	2	15
Kaufman, 2005	2	2	2	2	2	2	2	2	16
Zajac and Oertli et al., 2003	2	2	2	2	2	2	2	2	16

[$]The items are scored 0 (not reported), 1 (reported but inadequate) or 2 (reported and adequate).

[#]This is a randomized, open-label study, we only extracted and assessed data from the one arm in it.
